# Supplementary material for: Interchain supramolecular interactions drive nearly 21% efficiency organic solar cells
Source: Nat Commun. 2026 Mar 30;17:4590. doi: 10.1038/s41467-026-71199-0 (PMC13195077; doi:10.1038/s41467-026-71199-0)
Supplement: Supplementary file 1 — Supplementary Information [file 41467_2026_71199_MOESM1_ESM.pdf]

Supplementary Information

Interchain supramolecular interactions drive nearly 21% efficiency organic solar cells

Wei Gao[1,†], Yulong Hai[2,†], Jinyan Zeng[1], Hao Xia[1,*], Ruijie Ma[3,4,*], Top Archie Dela Peña[2], Jiaying Wu[2], Chunhui Duan[5], Jian-Xin Tang[6], Zhanhua Wei[1,*], Gang Li[3,*]

[1]Xiamen Key Laboratory of Optoelectronic Materials and Advanced Manufacturing, Institute of Luminescent Materials and Information Displays, College of Materials Science and Engineering, Huaqiao University, Xiamen 361021, China
[2]Thrust of Advanced Materials, The Hong Kong University of Science and Technology (Guangzhou), Nansha, Guangzhou 511453, China
[3]Department of Electrical and Electronic Engineering, Research Institute for Smart Energy (RISE), Photonic Research Institute (PRI), The Hong Kong Polytechnic University, Hong Kong 999077, China
[4]Hangzhou International Innovation Institute, Beihang University, Hangzhou 311115, China
[5]Institute of Polymer Optoelectronic Materials and Devices, Guangdong Basic Research Center of Excellence for Energy & Information Polymer Materials, State Key Laboratory of Luminescent Materials and Devices, South China University of Technology, Guangzhou 510640, China
[6]Macao Institute of Materials Science and Engineering (MIMSE), Faculty of Innovation Engineering, Macau University of Science and Technology, Taipa 999078, Macao, China
[†]These authors contributed equally
*Corresponding authors: xiahao919@hqu.edu.cn; ruijiema@buaa.edu.cn; weizhanhua@hqu.edu.cn; gang.w.li@polyu.edu.hk

Materials synthesis

All solvents and reagents were used as received from commercial sources and used without further purification except for toluene and tetrahydrofuran (THF) which were dried by solvent purification system. 4,7-dibromo-5,6-dinitrobenzo[*c*][1,2,5]thiadiazole and 2-(5,6-difluoro-3-oxo-2,3-dihydro-1*H*-inden-1-ylidene)malononitrile (IC-2F) were purchased from Hyper company. ^{1}H NMR and ^{13}C NMR spectra were recorded on Bruker 500 MHz NMR spectrometer. The matrix-assisted laser desorption time-of-flight mass spectrometer (MALDI-TOF-MS) were performed on Micorflex^{TM} LRF.

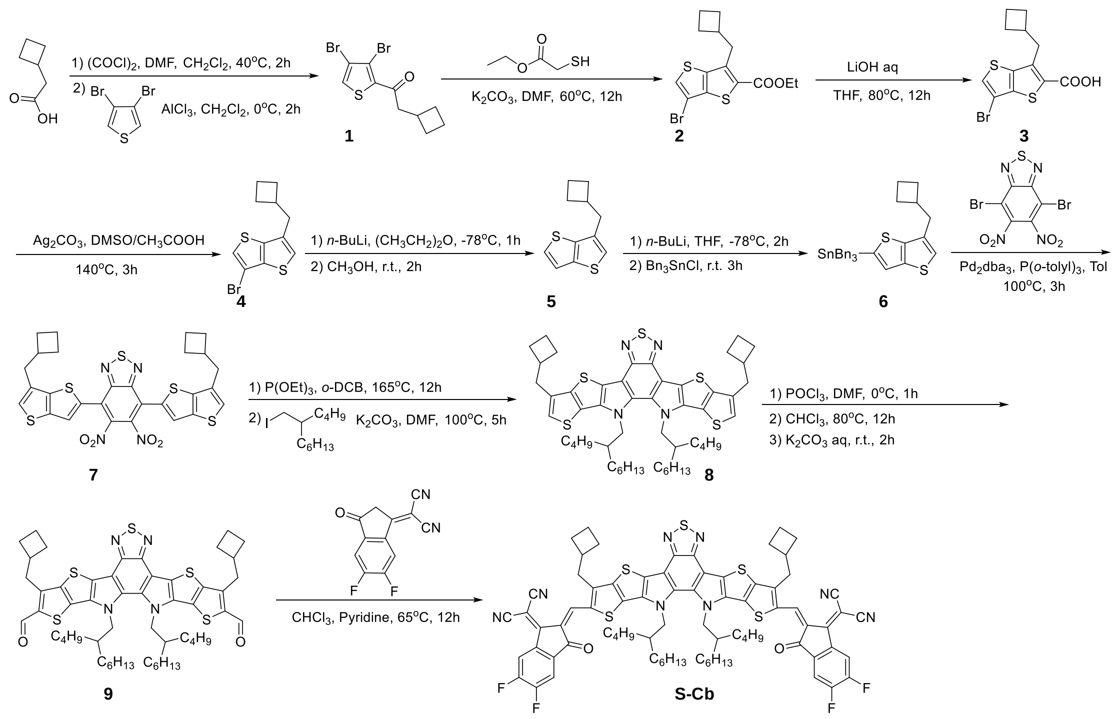

Supplementary Fig. 1 Synthetic routes of S-Ch.

Synthesis of 2-cyclobutyl-1-(3,4-dibromothiophen-2-yl)ethan-1-one (compound ***1****)*: To a 250 mL flask was added 2-cyclobutylacetic acid (5.0 g, 43.8 mmol), oxalyl chloride (6.1 g, 48.2 mmol) and anhydrous dichloromethane (DCM, 80 mL). Under nitrogen (N_2) protection, 1 mL dry *N*,*N*-dimethylformamide (DMF) was added dropwise to this stirring solution. After stirring at 40°C for two hours, the solvent was removed under reduced pressure. The residue was dissolved in 100 mL dry DCM and transferred to a 250 mL two-necked flask, and 3,4-dibromothiophene (10.6 g, 43.8 mmol) was added. Under 0°C and N_2 protection, $AlCl_3$ (7.0 g, 52.6 mmol) powered was added slowly in small batches. After stirring at room temperature for two hours, the mixture was poured into ice water and extracted with DCM, then washed with water and dried over anhydrous Na_2SO_4. After removal of the solvent under reduced pressure, the residue was purified by column chromatography on silica gel using a mixture solvent as eluent (hexane/dichloromethane, v/v = 2/1) to give a yellow solid (12.1 g, 82%). ^{1}H NMR (500 MHz, $CDCl_3$), δ (ppm): 7.60 (s, 1H), 3.18 (d, 2H), 2.86 (m, 1H), 2.20 (m, 2H), 1.91 (m, 2H), 1.74 (m, 2H). ^{13}C NMR (125 MHz, $CDCl_3$), δ (ppm): 191.15, 139.87, 129.30, 117.16, 117.12, 48.21, 31.68, 28.62, 19.08.

*Synthesis of ethyl 6-bromo-3-(cyclobutylmethyl)thieno[3,2-b]thiophene-2-carboxylate (compound **2**)*: To a 250 mL flask was added compound **1** (12.0 g, 35.5 mmol), potassium carbonate (K_2CO_3, 14.7 g, 107 mmol), ethyl 2-mercaptoacetate (4.3 g, 35.5 mmol), 18-crown-6 (0.25 g) and DMF (100 mL). Under N_2 protection, this mixture was allowed to keep stirring at 60°C for 12 hours. When the reaction was completed, water was added and extracted with DCM, then washed with water and dried over anhydrous Na_2SO_4. The residue was purified by column chromatography on silica gel using a mixture solvent as eluent (hexane/dichloromethane, v/v = 1/1) to give a yellow solid (11.3 g, 89%). ^{1}H NMR (500 MHz, $CDCl_3$), δ (ppm): 7.43 (s, 1H), 4.38 (q, 2H), 3.26 (d, 2H), 2.75-2.80 (m, 1H), 1.99-2.03 (m, 2H), 1.77-1.88 (m, 4H), 1.40 (t, 3H). ^{13}C NMR (125 MHz, $CDCl_3$), δ (ppm):162.93, 142.55, 142.03, 140.49, 128.97, 127.48, 103.22, 61.29, 36.14, 35.74, 28.65, 18.56, 14.45.

*Synthesis of 6-bromo-3-(cyclobutylmethyl)thieno[3,2-b]thiophene-2-carboxylic acid (compound **3**)*: To a 250 mL flask was added compound **2** (11.0 g, 30.6 mmol), THF (100 mL) and 3M LiOH aqueous solution (20 ml). The mixture was heated to 80°C and stirred overnight. After cooling to room temperature, THF was partially evaporated, and 10% HCl aqueous solution was dropwise added to acidify the solution (PH = 1), and then the precipitate was filtered and dried (9.0 g, 89%). ^{1}H NMR (500 MHz, $CDCl_3$), δ (ppm): 7.49 (s, 1H), 3.29 (d, 2H), 2.80 (m, 1H), 2.02 (m, 2H), 1.83 (m, 4H). ^{13}C NMR (125 MHz, $CDCl_3$), δ (ppm): 168.73, 144.51, 143.31, 140.67, 128.51, 128.00, 103.37, 36.20, 35.89, 28.72, 18.58.

*Synthesis of 3-bromo-6-(cyclobutylmethyl)thieno[3,2-b]thiophene (compound **4**)*: In a 100 mL flask, compound **3** (9.0 g, 27.2 mmol), Ag_2CO_3 power (750 mg, 2.72 mmol), dimethyl sulfoxide (40 ml), and acetic acid (2 ml) was added. The mixture was heated to 140°C and stirred for three hours. After cooling to room temperature, the mixture extracted with hexane and washed with water. The collected organic layer was dried over anhydrous Na_2SO_4 and concentrated. The residue was purified by column chromatography on silica gel using hexane to give a colorless liquid (6.5 g, 83%). ^{1}H NMR (500 MHz, $CDCl_3$), δ (ppm): 7.24 (s, 1H), 7.02 (s, 1H), 2.78 (m, 3H), 2.11 (m, 2H), 1.88 (m, 2H), 1.75 (m, 2H). ^{13}C NMR (125 MHz, $CDCl_3$), δ (ppm): 140.01, 139.39,

134.57, 123.44, 122.89, 103.08, 36.87, 35.23, 28.53, 18.42.

*Synthesis of 3-(cyclobutylmethyl)thieno[3,2-b]thiophene (compound **5**)*: To a stirring solution of compound **4** (6.4 g, 22.3 mmol) in dry ether (50 ml) was added dropwise a 2.4 M solution of *n*-butyllithium in hexane (10.2 mL, 24.5 mmol) at -78ºC under N_2 atmosphere. After stirring for 1 h, methanol (3 mL) was added, and then the mixture was allowed to stir at room temperature for two hours. After the reaction finished, the mixture was poured into water and extracted with hexane, then washed with water and dried over anhydrous Na_2SO_4. After removal of the solvent under reduced pressure, the residue was purified by column chromatography on silica gel using hexane to give a colorless liquid (4.1 g, 87%). ^{1}H NMR (500 MHz, $CDCl_3$), δ (ppm): 7.36 (d, 1H), 7.25 (d, 1H), 6.97 (s, 1H), 2.82 (m, 3H), 2.13 (m, 2H), 1.90 (m, 2H), 1.77 (m, 2H). ^{13}C NMR (125 MHz, $CDCl_3$), δ (ppm): 140.29, 138.73, 133.51, 126.65, 122.04, 120.04, 37.21, 35.23, 28.58, 18.46.

*Synthesis of tribenzyl(6-(cyclobutylmethyl)thieno[3,2-b]thiophen-2-yl)stannane (compound **6**)*: To a stirring solution of compound **5** (4.0 g, 19.2 mmol) in dry THF (30 ml) was added dropwise a 2.4 M solution of *n*-butyllithium in hexane (8.8 mL, 21.1mmol) at -78ºC under N_2 atmosphere. After stirring for 2 h, tributyltin chloride (6.9 g, 21.1 mmol) was added, and then the mixture was allowed to stir at room temperature for 3 h. After the reaction finished, the mixture was poured into water and extracted with hexane, then washed with water and dried over anhydrous Na_2SO_4. After removal of the solvent under reduced pressure, the product was collected and used without further purification.

Synthesis of 4,7-bis(6-(cyclobutylmethyl)thieno[3,2-b]thiophen-2-yl)-5,6-dinitrobenzo[c][1,2,5]thiadiazole (compound 7): To a dry 100 mL round-bottom flask, compound **6** (6.0 g, 10 mmol), 4,7-dibromo-5,6-dinitrobenzo[*c*][1,2,5]thiadiazole (1.3 g, 3.4 mmol), Pa_2dba_3 (122 mg, 0.14 mmol), P(*o*-tolyl)$_3$ (340 mg, 1.12 mmol) and anhydrous toluene (50 ml) was added under N_2 protection. Then, the mixture was refluxed at 100ºC for 3 h. After cooling to room temperature, the solvent was evaporated and the residue was purified by silicon chromatography using hexane/dichloromethane (4:1, v/v) as eluent to get the product as a red solid (1.6 g,

74%). ^{1}H NMR (500 MHz, $CDCl_3$), δ (ppm): 7.71 (s, 2H), 7.14 (s, 2H), 2.86 (d, 4H), 2.81 (m, 2H), 2.17 (m, 4H), 1.91 (m, 4H), 1.79 (m, 4H). ^{13}C NMR (125 MHz, $CDCl_3$), δ (ppm): 152.28, 144.57, 141.83, 139.13, 133.73, 130.16, 125.50, 124.23, 121.49, 37.04, 35.08, 28.58, 18.46. MALDI-TOF-MS m/z: [M] calcd. for $C_{28}H_{22}N_4O_4S_5$, 638.024, found 638.035.

*Synthesis of 12,13-bis(2-butyloctyl)-3,9-bis(cyclobutylmethyl)-12,13-dihydro-[1,2,5]thiadiazolo[3,4-e]thieno[2'',3'':4',5']thieno[2',3':4,5]pyrrolo[3,2-g]thieno[2',3':4,5]thieno[3,2-b]indole (compound **8**)*: To a dry 100 mL round-bottom flask, compound **7** (600 mg, 0.94 mmol), triethyl phosphite (2 ml) and anhydrous *o*-dichlorobenzene (20 ml) was added under N_2 protection. Then, the mixture was heated to 165°C and kept stirring for 12h. After cooling to room temperature, the solvent was evaporated under reduced pressure. Without further purification, potassium carbonate (1.3 g, 9.4 mmol), 2-butyloctyl iodine (1.2 g, 4 mmol) and *N*,*N*-dimethylformamide (20 mL) were added into the residue. The mixture was kept stirring at 100°C for 5h. After cooling to room temperature, the mixture was extracted with dichloromethane and washed with water. The collected organic layer was dried over anhydrous Na_2SO_4 and concentrated. The residue was purified by silicon chromatography using hexane/dichloromethane (8:1, v/v) as eluent to get the product as a yellow solid (527 mg, 61%). ^{1}H NMR (500 MHz, $CDCl_3$), δ (ppm): 6.98 (s, 2H), 4.59 (d, 4H), 2.91 (m, 6H), 2.17 (m, 4H), 2.06 (m, 2H), 1.92 (m, 4H), 1.83 (m, 4H), 0.77-1.08 (m, 30H), 0.57-0.68 (m, 14H). ^{13}C NMR (125 MHz, $CDCl_3$), δ (ppm): 147.77, 142.39, 137.17, 137.15, 135.43, 131.80, 131.78, 123.69, 122.82, 122.79, 119.56, 111.61, 55.11, 55.08, 38.75, 36.73, 35.41, 31.69, 30.50, 30.40, 30.36, 29.47, 29.44, 28.66, 25.41, 25.19, 22.84, 22.58, 22.55, 18.49, 14.08, 14.06, 13.87, 13.83. MALDI-TOF-MS m/z: [M] calcd. for C52H70N4S5, 910.420, found 910.707.

*Synthesis of 12,13-bis(2-butyloctyl)-3,9-bis(cyclobutylmethyl)-12,13-dihydro-[1,2,5]thiadiazolo[3,4-e]thieno[2'',3'':4',5']thieno[2',3':4,5]pyrrolo[3,2-g]thieno[2',3':4,5]thieno[3,2-b]indole-2,10-dicarbaldehyde (compound **9**)*: To a dry 100 mL round-bottom flask, 5 ml anhydrous *N*, *N*-dimethylformamide (DMF) was added, and the solution was cooled to 0°C and stirred when 1 mL phosphorous

oxychloride ($POCl_3$) was added by syringe under N_2 protection. The mixture was stirred at 0°C for 1 hour, and then compound **8** (500 mg, 0.55 mmol) in dry chloroform (10 ml) was added. Then, the mixture solution was allowed to reflux overnight. After cooling to room temperature, K_2CO_3 aqueous solution was added and stirred for another 2 h ,and the mixture was extracted with dichloromethane (DCM), and the organic layer was collected, washed with water and dried with anhydrous Na_2SO_4. After removal of the solvent under reduced pressure, the residue was purified by column chromatography on silica gel using a mixture solvent as eluent (hexane/dichloromethane, v/v = 1/1) to give an orange solid (412 mg, 77%). ^{1}H NMR (500 MHz, $CDCl_3$), δ (ppm): 10.14 (s, 1H), 4.62 (d, 4H), 3.29 (d, 4H), 2.93 (m, 2H), 2.13 (m, 4H), 2.03 (m, 2H), 1.90 (m, 8H), 0.79-1.08 (m, 30 H), 0.58-0.69 (m, 14H). ^{13}C NMR (125 MHz, $CDCl_3$), δ (ppm): 181.96, 147.57, 145.30, 143.47, 137.55, 136.90, 136.89, 133.00, 132.98, 129.72, 127.45, 127.41, 112.49, 55.36, 55.33, 38.95, 38.94, 36.80, 34.93, 31.59, 30.38, 30.30, 30.26, 29.40, 29.38, 28.75, 27.97, 27.81, 25.32, 25.13, 22.78, 22.53, 22.51, 18.33, 14.03, 14.02, 13.81, 13.77. MALDI-TOF-MS m/z: [M] calcd. for $C_{54}H_{70}N_4O_2S_5$, 966.410, found 966.407.

*Synthesis of 2,2'-((2Z,2'Z)-((12,13-bis(2-butyloctyl)-3,9-bis(cyclobutylmethyl)-12,13-dihydro-[1,2,5]thiadiazolo[3,4-e]thieno[2",3":4',5']thieno[2',3':4,5]pyrrolo[3,2-g]thieno[2',3':4,5]thieno[3,2-b]indole-2,10-diyl)bis(methaneylylidene))bis(5,6-difluoro-3-oxo-2,3-dihydro-1H-indene-2,1-diylidene))dimalononitrile (**S-Cb**)*: To a 100 mL round bottom flask, compound **9** (200 mg, 0.21 mmol) and 2-(5,6-difluoro-3-oxo-2,3-dihydro-1*H*-inden-1-ylidene)malononitrile (190 mg, 0.83 mmol) were added under N_2 protection. Then, deoxidized chloroform (30 ml) was added and stirred for a while when pyridine (1 ml) was added. The mixture was kept stirring at 65°C for 12 h. After removal of chloroform of reaction mixture under reduced pressure, 100 ml methanol was added and the precipitate was collected by filtration. The residue was purified by column chromatography on silica gel using a mixture solvent as eluent (hexane/dichloromethane, v/v = 2/3) to give a dark solid (235 mg, 81%). ^{1}H NMR (300 MHz, $CDCl_3$), δ (ppm): 9.13 (s, 2H), 8.53 (t, 2H), 7.70 (t, 2H), 4.77 (d, 4H), 3.29 (d,

4H), 2.88 (m, 2H), 2.09 (m, 6H), 1.93 (m, 8H), 0.89-1.20 (m, 30 H), 0.64-0.71 (m, 14H). MALDI-TOF-MS m/z: [M] calcd. for $C_{78}H_{74}F_4N_8O_2S_5$, 1390.447, found 1390.448.

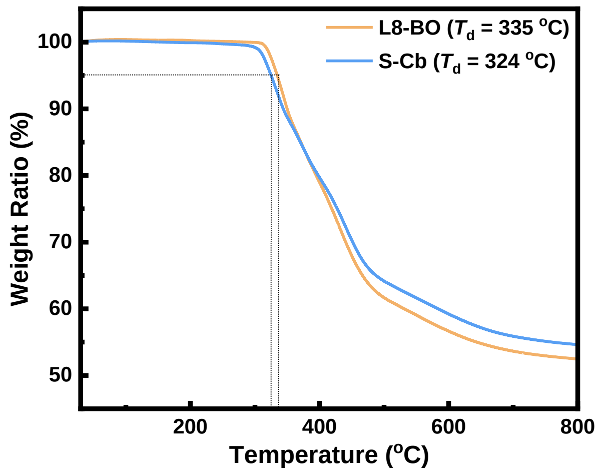

Supplementary Fig. 2 TGA curves of S-Cb and L8-BO.

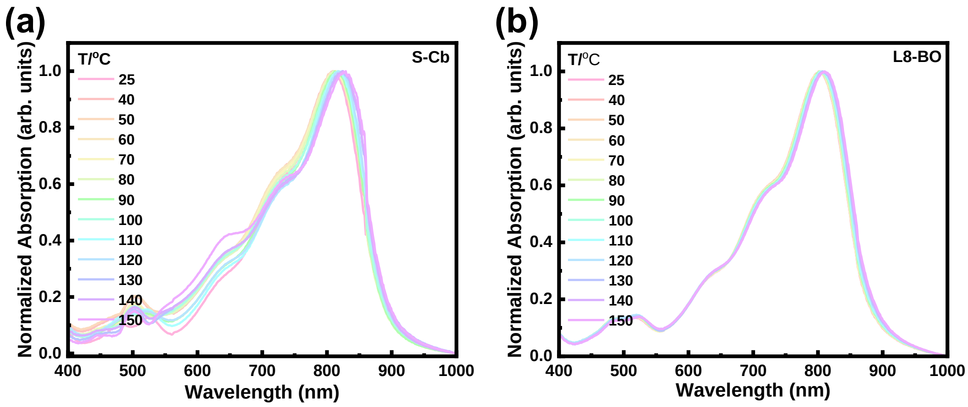

Supplementary Fig. 3 Temperature-dependent UV-vis absorption spectra of (a) S-Cb and (b) L8-BO neat films.

Supplementary Table 1 Absorption and energy level properties of S-Cb and L8-BO.

Acceptor	λ_{max}[a)] (nm)	λ_{onset}[a)] (nm)	ε_{Max} (M^{-1} cm^{-1})	λ_{max}[b)] (nm)	λ_{onset}[b)] (nm)	ε_{Max} (cm^{-1})	E_g^{opt}[c)] (eV)
L8-BO	731	787	2.04×10^5	800	896	3.97×10^4	1.384
S-Cb	733	789	2.15×10^5	803	913	4.49×10^4	1.358

[a)]In solution. [b)]In thin film. [c)]Calculated from $E_g^{opt} = 1240/\lambda_{onset}$.

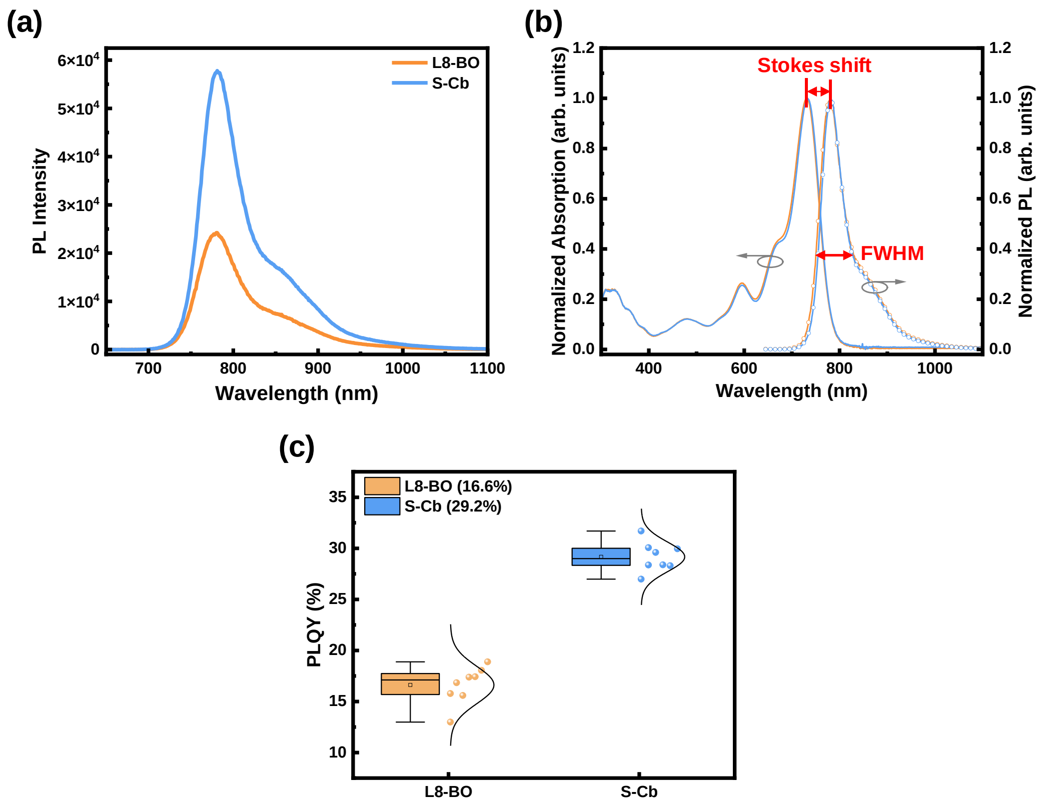

Supplementary Fig. 4 (a) PL spectra of L8-BO and S-Cb in dilute solution with the same concentration. (b) Normalized absorption and PL spectra of L8-BO and S-Cb in solution. (c) PLQY results of L8-BO and S-Cb in solution (the box plot elements are defined as follows: the center line represents the median; box limits represent the first (Q1) and third (Q3) quartiles; whiskers represent the maximum and minimum values within 1.5 times the interquartile range (IQR); the small square represents the mean value; the error bar represents the standard error of the mean with n = 8; individual data points show each measurement).

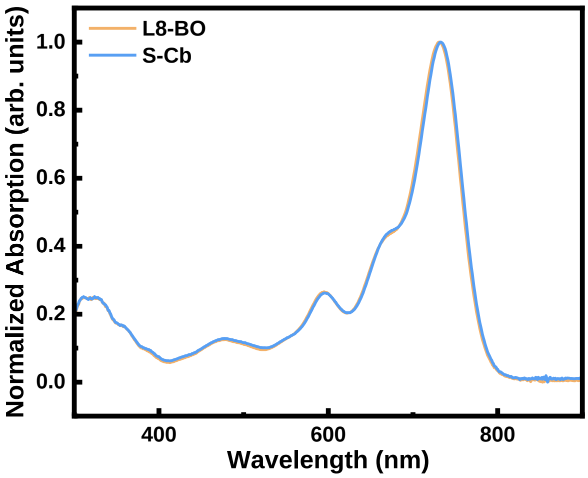

Supplementary Fig. 5 Normalized UV-Vis absorption spectra of L8-BO and S-Cb in diluted chloroform solution.

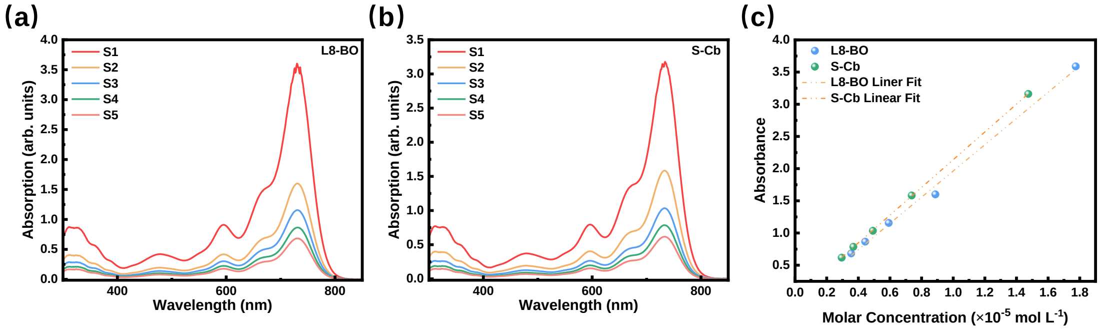

Supplementary Fig. 6 (a) UV-Vis absorption spectra of L8-BO at different concentrations. (b) UV-Vis absorption spectra of S-Cb at different concentrations. (c) Plots of maximum absorbance versus concentration for L8-BO and S-Cb, along with the corresponding linear fits.

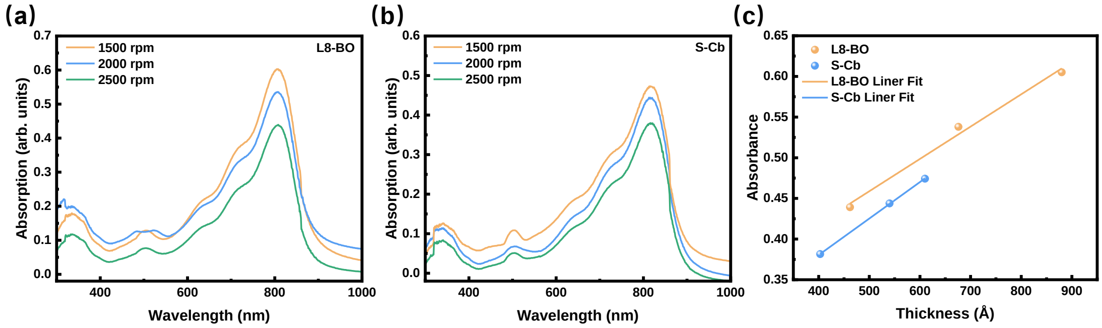

Supplementary Fig. 7 (a) UV-Vis absorption spectra of L8-BO under different thickness. (b) UV-Vis absorption spectra of S-Cb under different thickness. (c) Plots of maximum absorbance versus thickness for L8-BO and S-Cb films, along with the

corresponding linear fits.

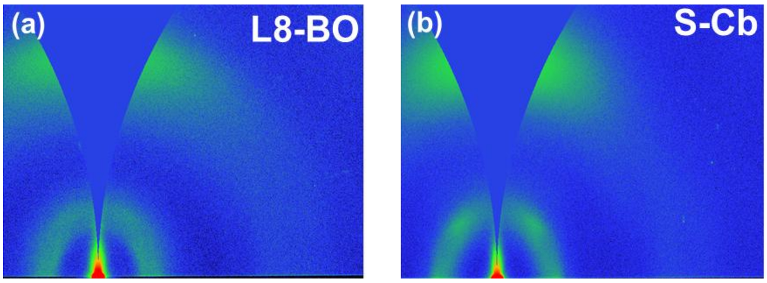

Supplementary Fig. 8 2D GIWAXS patterns for (a) L8-BO and (b) S-Cb.

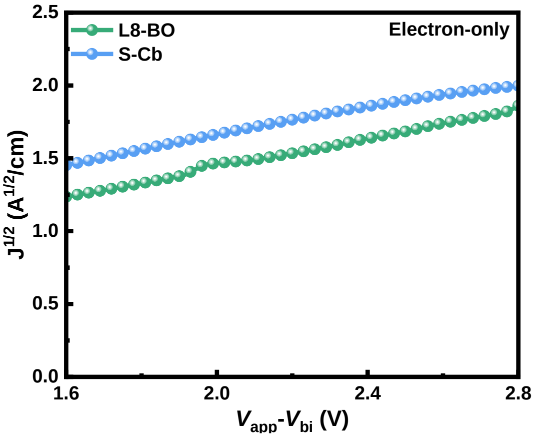

Supplementary Fig. 9 *J*-*V* characteristics obtained from SCLC measurements of neat S-Cb and L8-BO films.

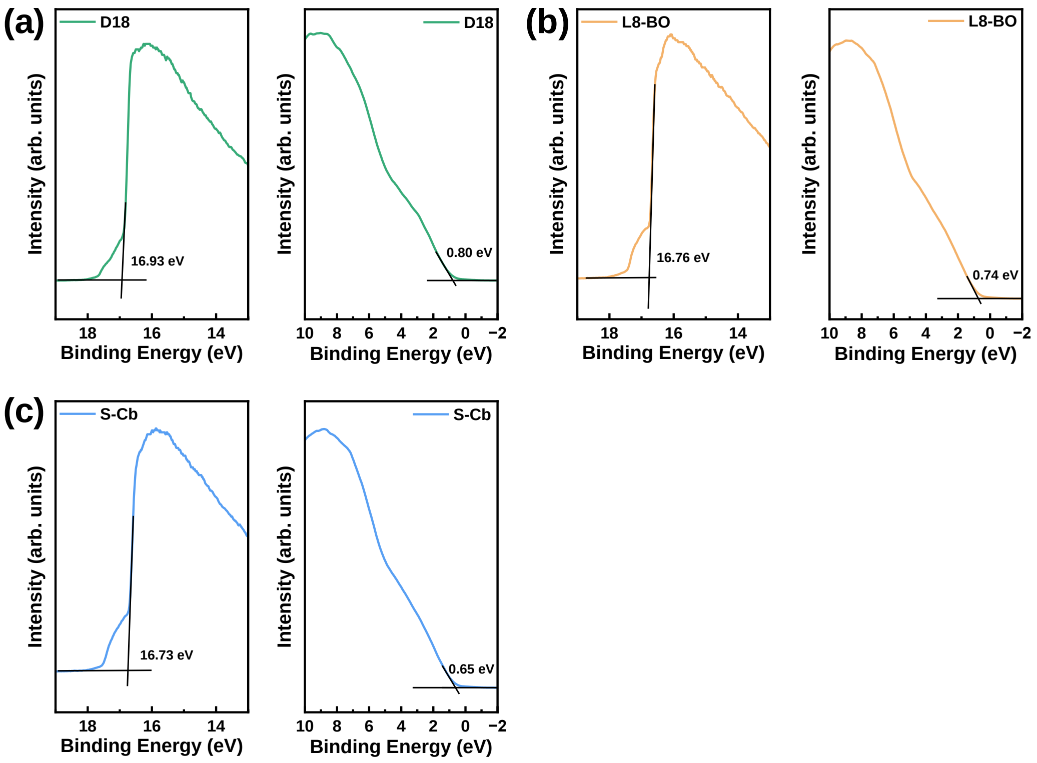

Supplementary Fig. 10 UPS measurements for (a) D18, (b) L8-BO, and (c) S-Cb.

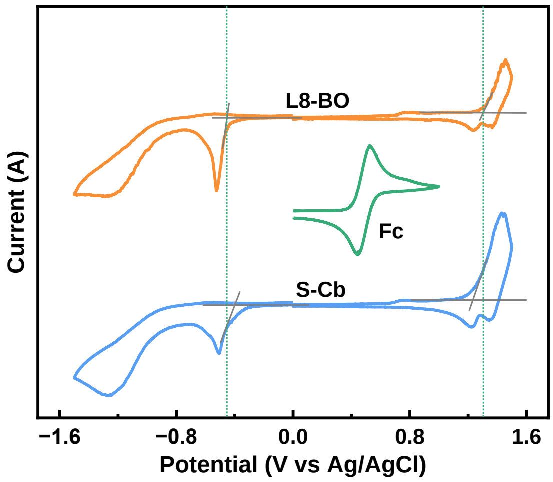

Supplementary Fig. 11 CV curves for L8-BO and S-Cb.

华南国家计量测试中心
广东省计量科学研究院
SOUTH CHINA NATIONAL CENTER OF METROLOGY
GUANGDONG INSTITUTE OF METROLOGY

校 准 结 果
RESULTS OF CALIBRATION

证书编号 NYX202400281 原始记录号 NYX202400281 第 3 页,共 4 页
Certificate No. Record No. Page of

一、外观检查： 符合要求
Apparent Inspecti Pass.

二、测试条件： 温度(25±2)℃；辐照度1000W/m^2。
Test conditions: Temperature: (25±2)℃；Irradiance：1000W/m^2.

三、电流-电压曲线：
The IV curve:

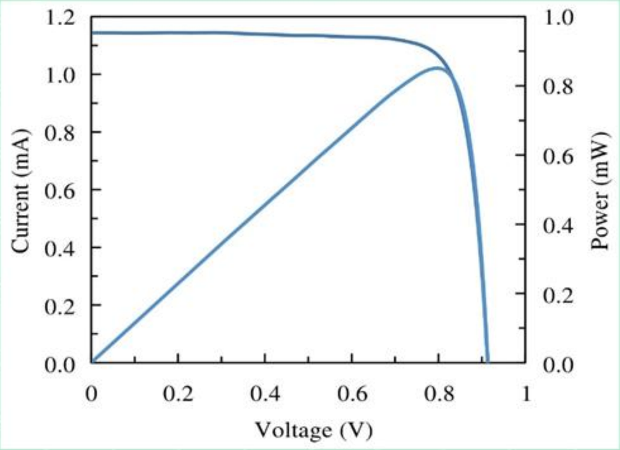

四、光电性能参数：
Results of photoelectric properties:

表1(Table 1)

短路电流密度J_{sc} Short ciruit current density	短路电流 I_{sc} Short ciruit current	开路电压 V_{oc} Open circuit voltage	填充因子 *FF* Fill factor	最大功率 P_m Maximum power	最佳工作电流I_m Optimum working current	最佳工作电压V_m Optimum working voltage	转换效率 η Efficiency
mA/cm^2	mA	V	%	mW	mA	V	%
27.90	1.144	0.913	81.4	0.850	1.063	0.800	20.74

Supplementary Fig. 12 Efficiency certification report.

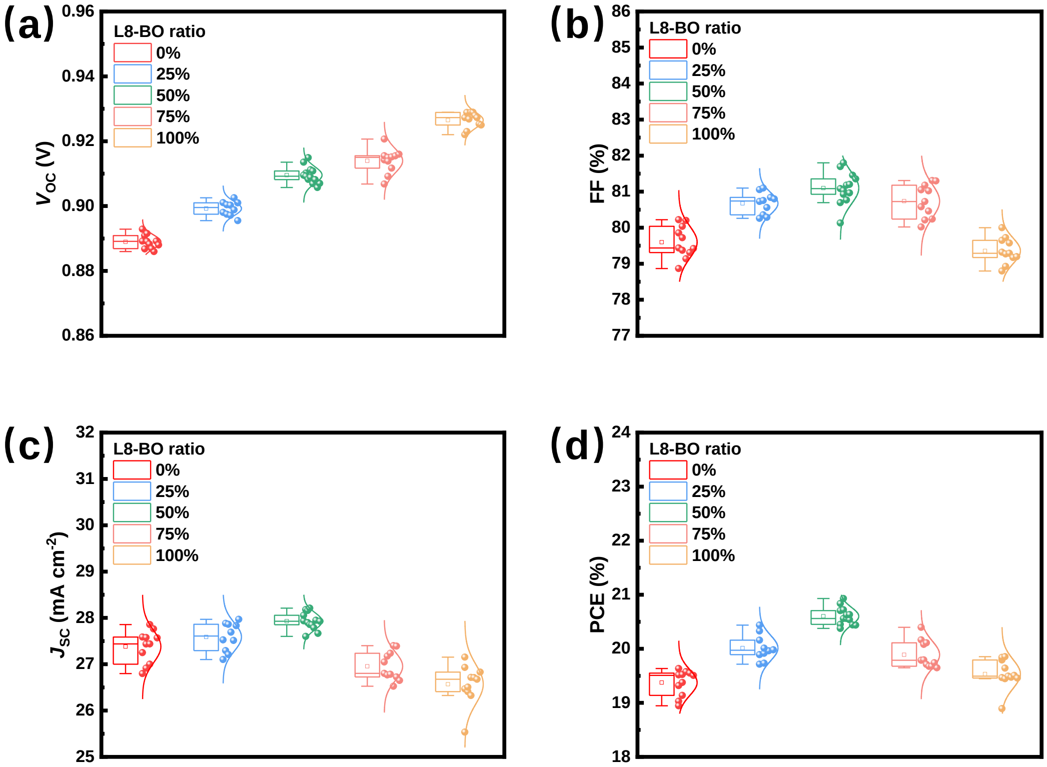

Supplementary Fig. 13 Photovoltaic parameters of 10 to 15 OSC devices based on D18:S-Cb:L8-BO with varying L8-BO ratios: (a) V_{OC}, (b) FF, (c) J_{SC}, and (d) PCE. The box plot elements are defined as follows: the center line represents the median; box limits represent the first (Q1) and third (Q3) quartiles; whiskers represent the maximum and minimum values within 1.5 times the interquartile range (IQR); the small square represents the mean value; the error bar represents the standard error of the mean; individual data points show each measurement

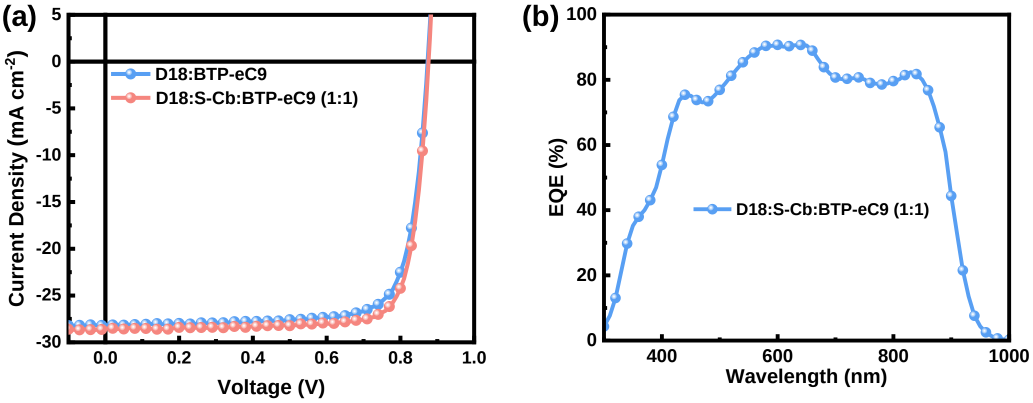

Supplementary Fig. 14 (a) Optimal *J-V* curves for OSCs based on D18:BTP-eC9 and D18:S-Cb:BTP-eC9 (1:1). (b) Corresponding EQE spectrum for OSCs based on D18:S-

Cb:BTP-eC9 (1:1).

Supplementary Table 2 Photovoltaic parameters of optimized OSCs based on D18:BTP-eC9 and D18:S-Cb:BTP-eC9 (1:1).

BTP-eC9 ratio	V_{oc} (V)	J_{sc} (mA cm^{-2})	J_{sc}[a)] (mA cm^{-2})	FF (%)	PCE[b)] (%)
50%	0.876	28.54	27.10	80.9	20.23 (20.13 ± 0.09)
100%[c)]	0.873	28.17	-	78.0	19.21 (19.06 ± 0.15)

[a)]The integrated J_{SC} were calculated from the EQE spectra.

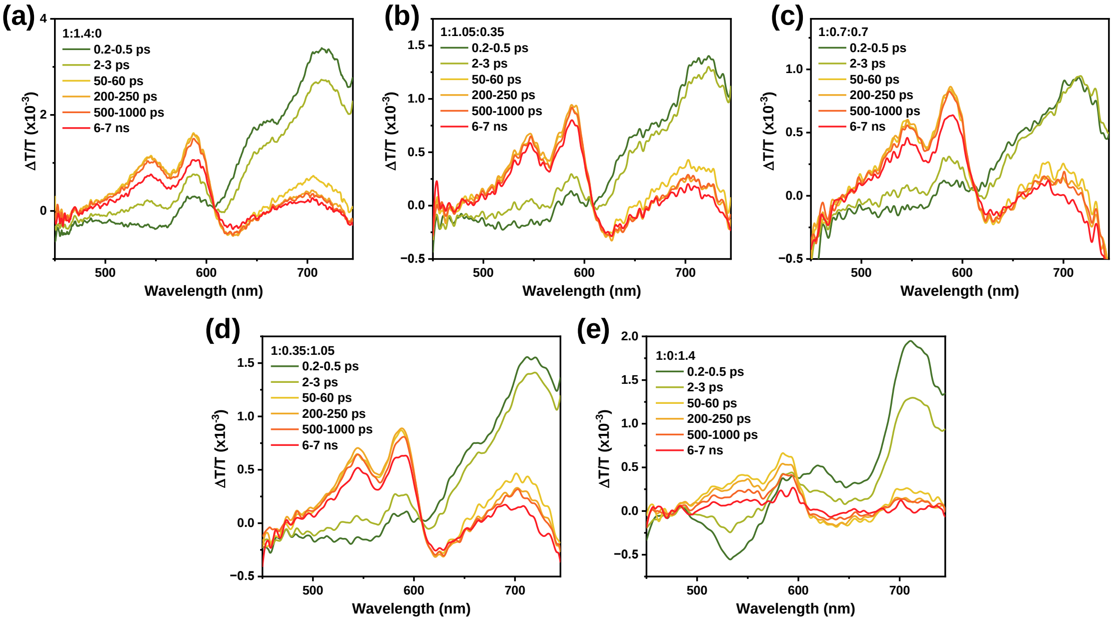

Supplementary Fig. 15 Spectral cuts at representative pump-probe delay times for (a) D18:S-Cb:L8-BO with 0% L8-BO; (b) D18:S-Cb:L8-BO with 25% L8-BO; (c) D18:S-Cb:L8-BO with 50% L8-BO; (d) D18:S-Cb:L8-BO with 75% L8-BO; (e) D18:S-Cb:L8-BO with 100% L8-BO.

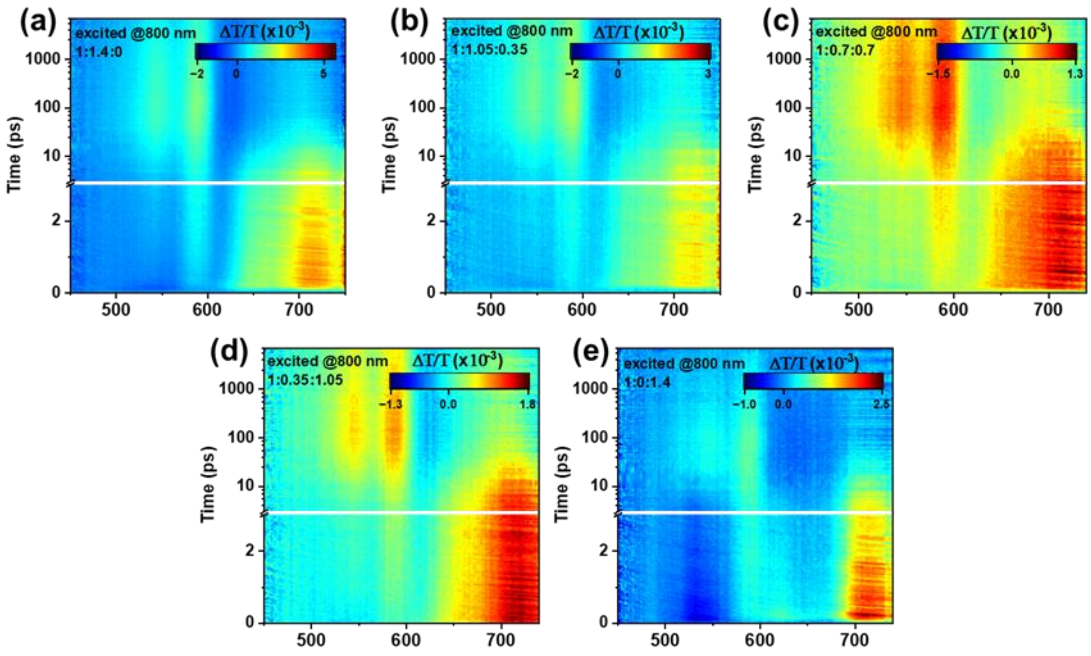

Supplementary Fig. 16 Corresponding 2D TAS contour plots for (a) D18:S-Cb:L8-BO with 0% L8-BO; (b) D18:S-Cb:L8-BO with 25% L8-BO; (c) D18:S-Cb:L8-BO with 50% L8-BO; (d) D18:S-Cb:L8-BO with 75% L8-BO; (e) D18:S-Cb:L8-BO with 100% L8-BO.

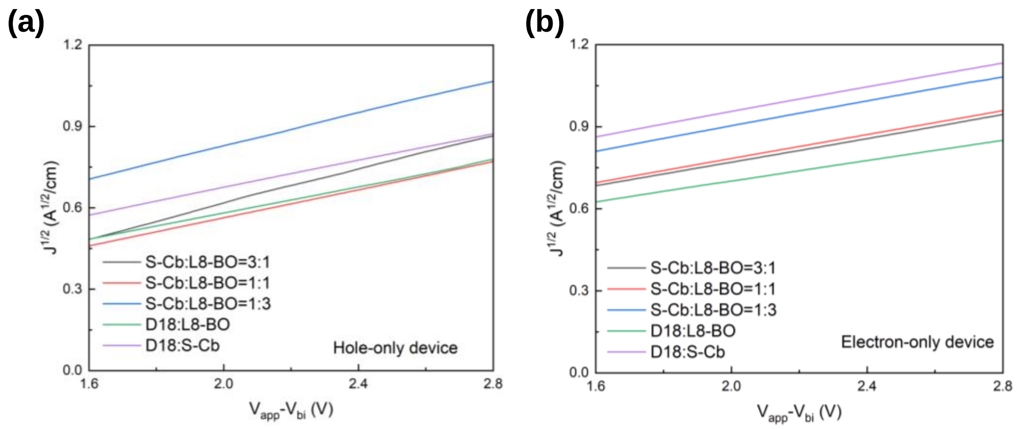

Supplementary Fig. 17 *J-V* characteristics obtained from SCLC measurements of D18:S-Cb:L8-BO active layer with different L8-BO content: (a) for hole-only devices and (b) for electron-only devices.

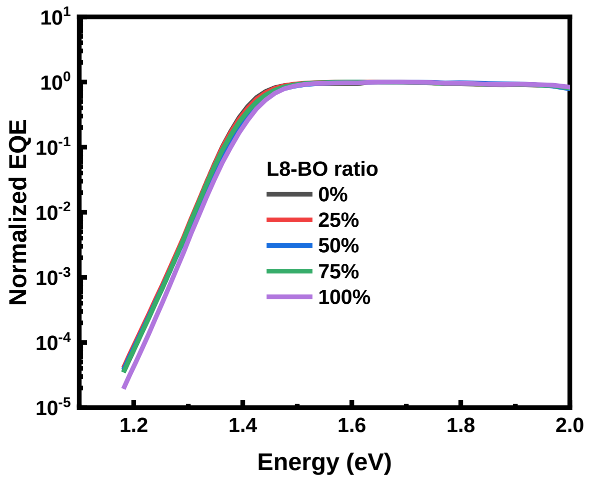

Supplementary Fig. 18 Fourier transform photonic spectroscopy EQE of OSCs based on D18:S-Cb:L8-BO with 0%, 25%, 50%, 75% and 100% L8-BO content.

Supplementary Table 3 Energy loss data of D18:S-Cb:L8-BO-based OSCs with 0%, 25%, 50%, 75% and 100% L8-BO contents.

L8-BO ratio	E_{gap} [eV]	V_{oc} [V]	E_{loss} [eV]	$\Delta E_1 = E_{gap} - qV_{oc}^{SQ}$ [eV]	$\Delta E_2 = q\Delta V_{oc}^{rad}$ [eV]	$\Delta E_3 = q\Delta V_{oc}^{non-rad}$ [eV]
0%	1.411	0.888	0.523	0.255	0.046	0.222
25%	1.418	0.901	0.517	0.255	0.045	0.217
50%	1.422	0.907	0.515	0.256	0.049	0.210
75%	1.428	0.916	0.512	0.256	0.027	0.229
100%	1.445	0.925	0.520	0.256	0.034	0.230

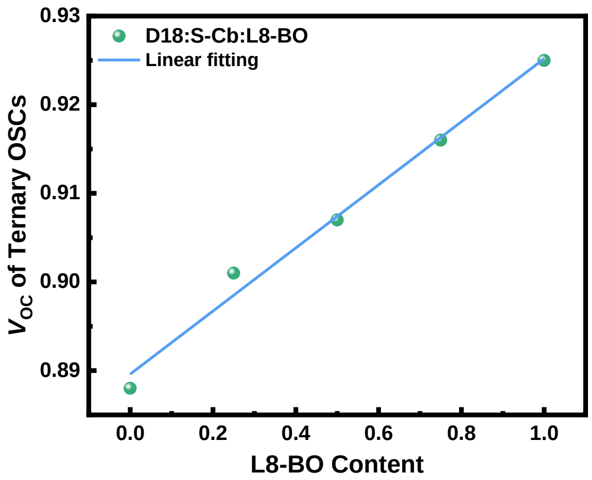

Supplementary Fig. 19 A linear increase of V_{OC}s of ternary OSCs based D18:S-Cb:L8-BO with the L8-BO content.

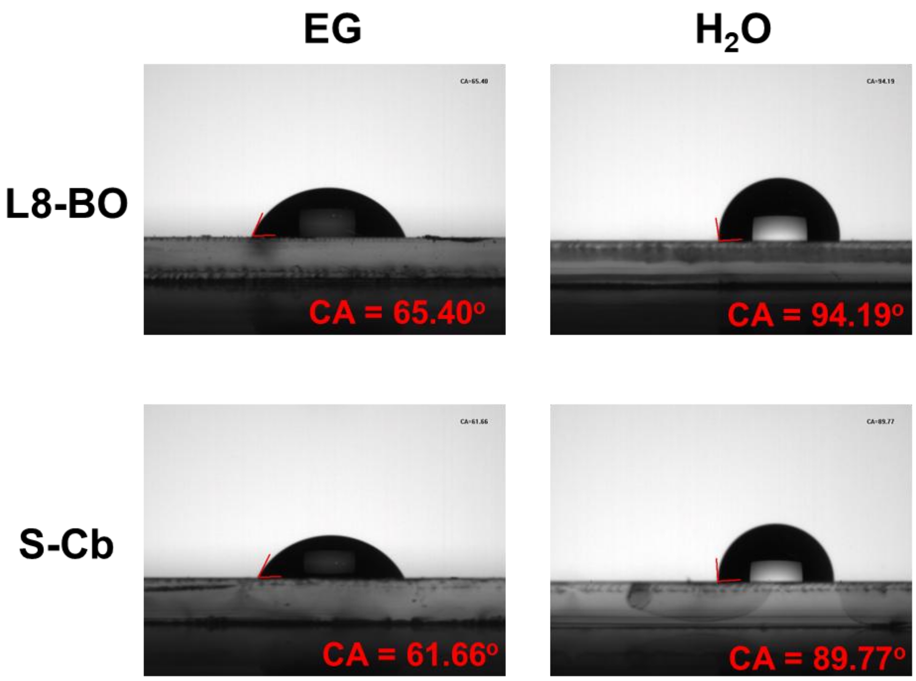

Supplementary Fig. 20 Contact angle measurement of L8-BO and S-Cb neat films.

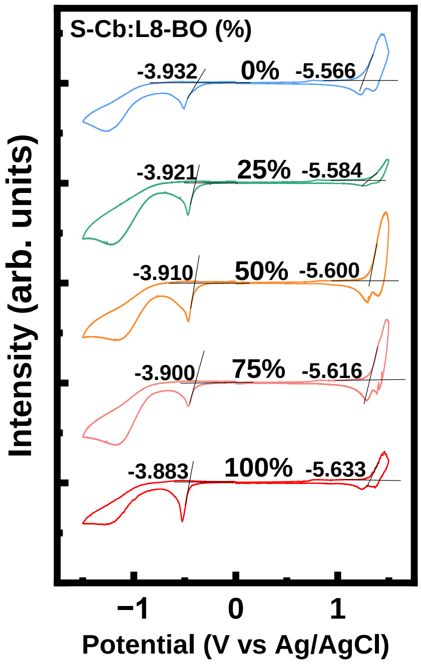

Supplementary Fig. 21 CV curves of S-Cb:L8-BO blend films with different L8-BO contents.

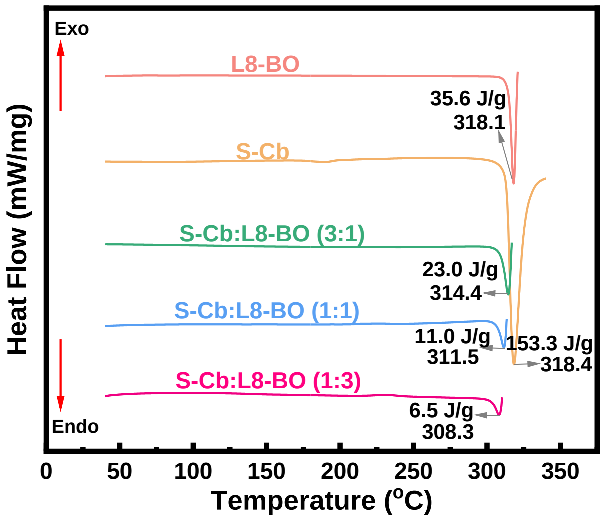

Supplementary Fig. 22 DSC curves of S-Cb:L8-BO blend films with different L8-BO contents.

Supplementary Table 4 The crystallographic parameters of S-Cb.

CCDC number	2423862
Empirical formula	$C_{78}H_{76}F_4N_8O_2S_5$
Formula weight	1393.81
Temperature [K]	100.00(10)
Crystal system	triclinic
Space group (number)	$P\bar{1}$ (2)
a [Å]	15.7544(4)
b [Å]	19.7276(4)
c [Å]	28.2326(7)
α [°]	99.645(2)
β [°]	99.733(2)
γ [°]	95.755(2)
Volume [Å^3]	8451.0(4)
Z	4
ρ_{calc} [gcm^{-3}]	1.050
μ [mm^{-1}]	1.682
F(000)	2792
Crystal size [mm^3]	0.21×0.14×0.12
Crystal colour	black
Crystal shape	prism
Radiation	Cu K_α (λ=1.54184 Å)
2θ range [°]	4.58 to 155.28 (0.79 Å)

Index ranges	$-19 \le h \le 19$
	$-24 \le k \le 20$
	$-34 \le l \le 35$
Reflections collected	98903
Independent reflections	32638
	$R_{int} = 0.0661$
	$R_{sigma} = 0.0605$
Completeness to	98.1 %
θ = 67.684°	
Data / Restraints / Parameters	32638/1032/1528
Goodness-of-fit on F^2	0.978
Final R indexes	$R_1 = 0.1488$
[$I \geq 2\sigma(I)$]	$wR_2 = 0.3979$
Final R indexes	$R_1 = 0.1791$
[all data]	$wR_2 = 0.4220$
Largest peak/hole [eÅ^{-3}]	1.17/-0.92

Definition of δG

In the independent gradient model based on Hirshfeld partition (IGMH) analysis[1], the atomic densities involved in definition of δg is derived based on Hirshfeld partition, namely

$$\rho_i^{\text{Hirsh}} = \rho(\boldsymbol{r})\boldsymbol{w}_i(\boldsymbol{r})$$

where ρ is the electron density of the whole system calculated based on wavefunction, and the Hirshfeld weighting function of atom i is expressed as

$$\boldsymbol{w}_i(\boldsymbol{r}) = \frac{\rho_i^{free}(\boldsymbol{r})}{\rho^{pro}(\boldsymbol{r})} = \frac{\rho_i^{free}(\boldsymbol{r})}{\sum_j \rho^{pro}(\boldsymbol{r})}$$

where ρ_i^{free} is spherically averaged electron density of atom i in its free state, ρ^{pro} corresponds to promolecular density, the index j loops over all atoms.

For three-dimensional cases, g^{IGM} and δg can be defined as follows.

$$g(\boldsymbol{r}) = \left|\sum_i \nabla \rho_i^{free}(\boldsymbol{r})\right|$$

$$g^{IGM}(\boldsymbol{r}) = \sum \left|\nabla \rho_i^{free}(\boldsymbol{r})\right|$$

$$\delta g(\boldsymbol{r}) = g^{IGM}(\boldsymbol{r}) - g(\boldsymbol{r})$$

The ρ_i^{free} stands for spherically averaged density of atom i in its free state.

The atomic pair δg index (δGpair) was defined to quantify the contribution of atomic pair to the interaction between two fragments (A and B)

$$\delta G_{i,j}^{pair} = \int \delta g_{i,j}(\boldsymbol{r}) d\boldsymbol{r} = \int \left[g_{i,j}^{IGM}(\boldsymbol{r}) - g_{i,j}(\boldsymbol{r}) \right] d\boldsymbol{r} \quad \boldsymbol{i} \in \boldsymbol{A}, \boldsymbol{j} \in \boldsymbol{B}$$

where

$$g_{i,j}(\boldsymbol{r}) = \left| \nabla \rho_i^{free}(\boldsymbol{r}) + \nabla \rho_j^{free}(\boldsymbol{r}) \right|$$

$$g_{i,j}^{IGM}(\boldsymbol{r}) = \left| \nabla \rho_i^{free}(\boldsymbol{r}) + \nabla \rho_j^{free}(\boldsymbol{r}) \right|$$

It is also useful to define percentage atomic pair contribution to interfragmentary interaction as

$$\delta G_{i,j}^{pair}(\%) = \frac{\delta G_{i,j}^{pair}}{\sum_{k \in A} \sum_{l \in B} \delta G_{k,l}^{pair}}$$

It is able to accurately represent contribution of atomic pairs to interaction energy between two fragments, however δGpair(%) should be able to identify "hot" atomic pairs, which may indeed have large actual contribution to interfragmentary binding.

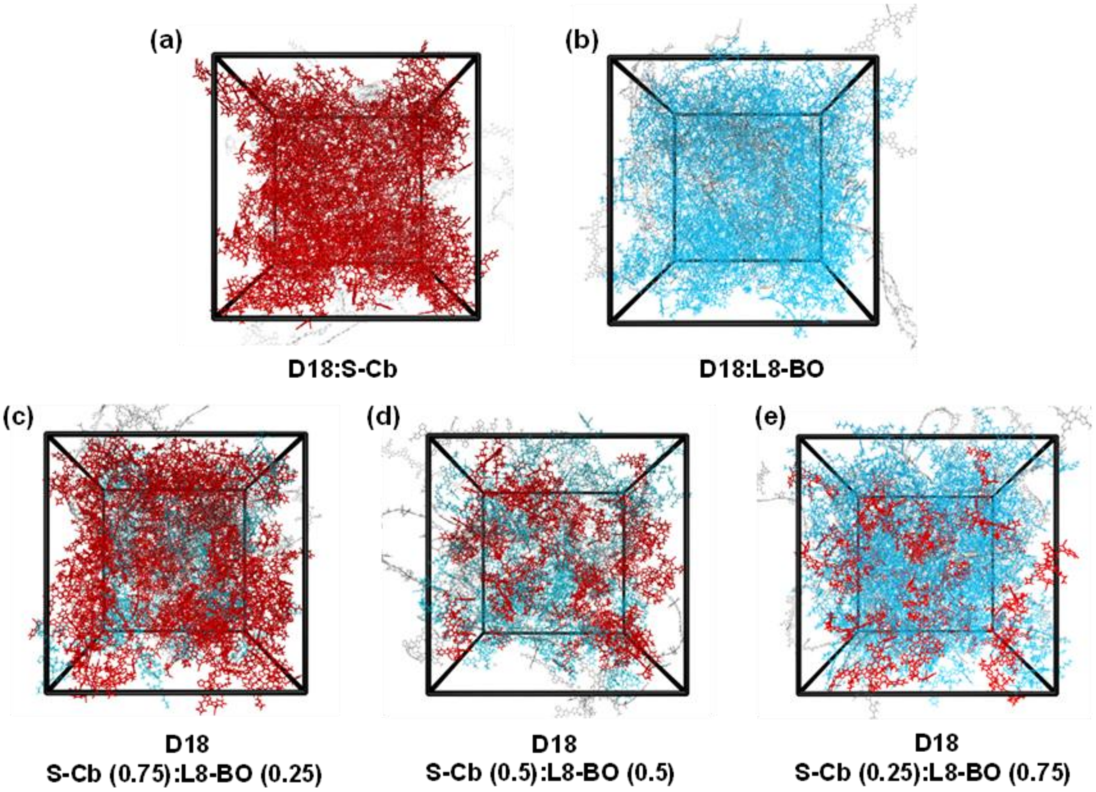

Supplementary Fig. 23 Molecular dynamics simulations to change different ratios of D18:S-Cb:L8-BO to simulate the formation of real blend films.

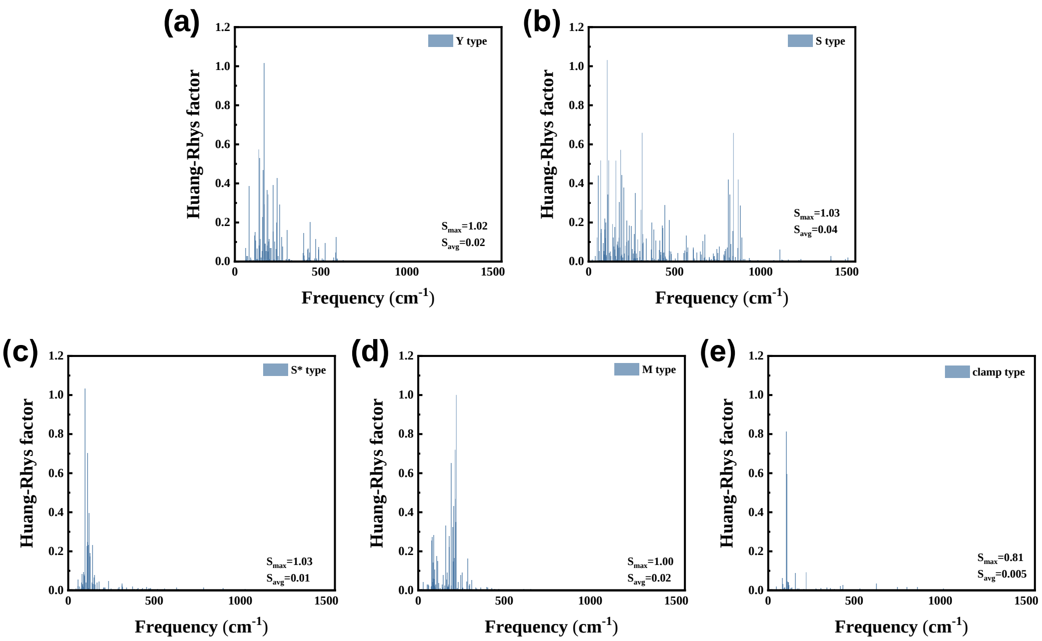

Supplementary Fig. 24 Huang-Rhys factor for (a) Y-type dimer of S-Cb; (b-d) S-, S*-, and M-type dimers of L8-BO; (e) clamp type dimer between S-Cb and L8-BO.

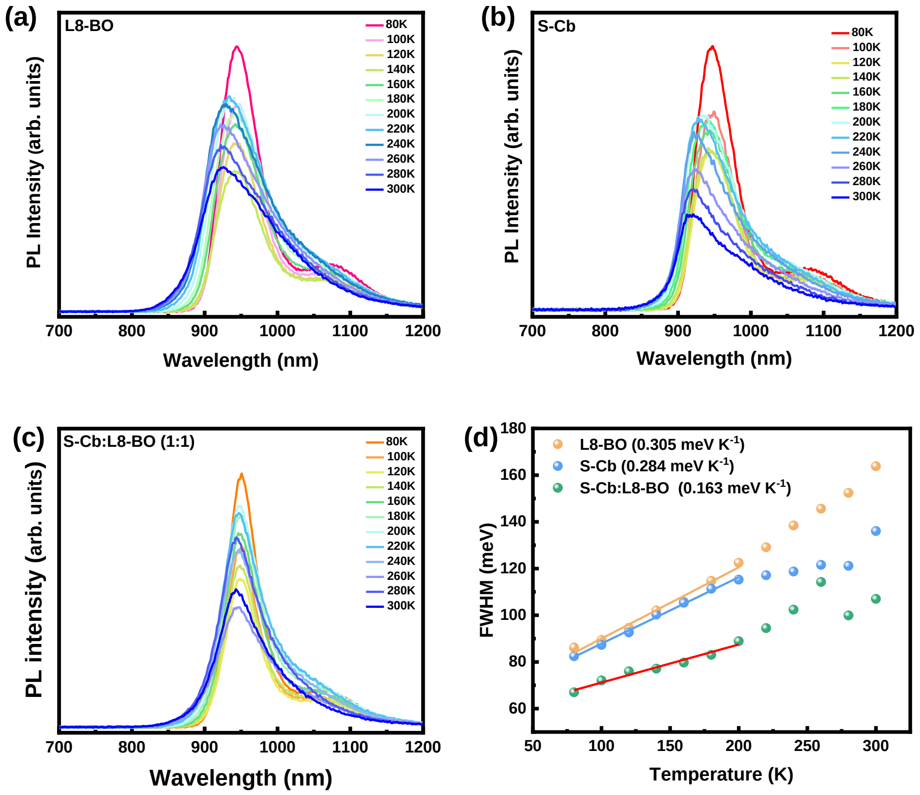

Supplementary Fig. 25 Temperature-dependent PL spectra of (a) L8-BO neat film, (b) S-Cb neat film, and (c) S-Cb:L8-BO (1:1) blend film. (d) Linear fitting in the low-temperature region between temperature and FWHM.

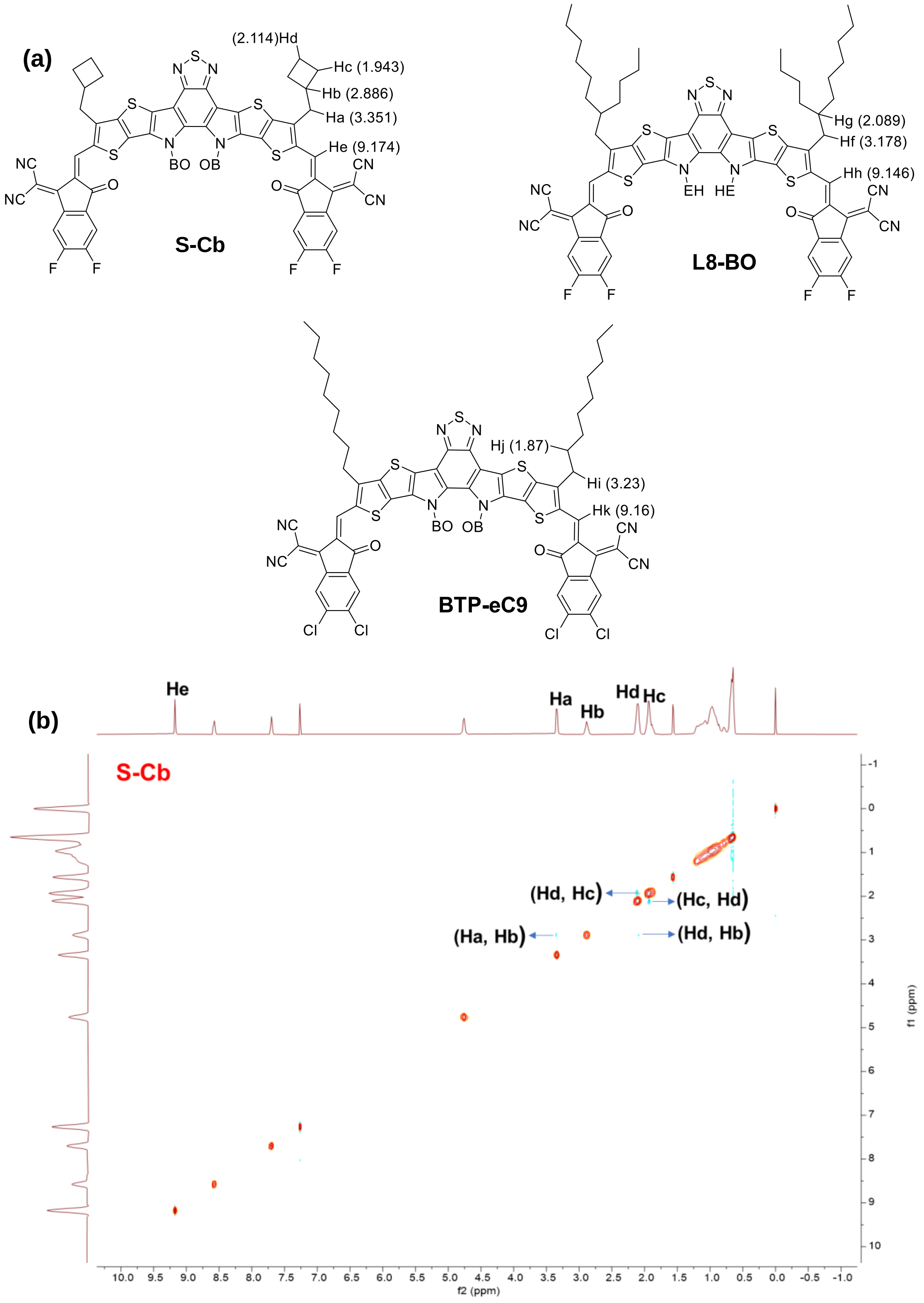

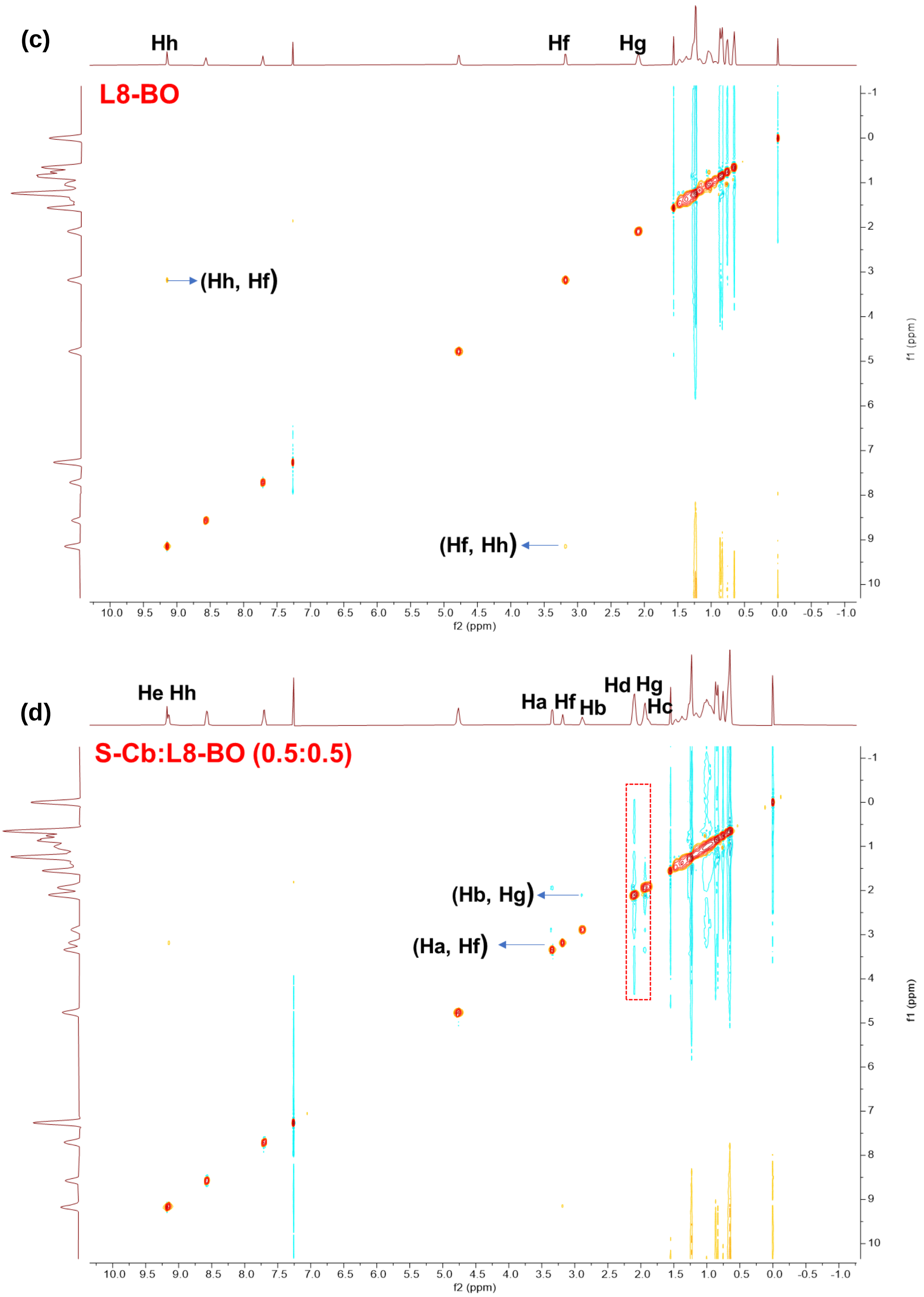

(c)
Hh
Hf
Hg
L8-BO
(Hh, Hf)
(Hf, Hh)
f2 (ppm)
f1 (ppm)
(d)
He Hh
Ha Hf Hb
Hd Hg
Hc
S-Cb:L8-BO (0.5:0.5)
(Hb, Hg)
(Ha, Hf)
f2 (ppm)
f1 (ppm)

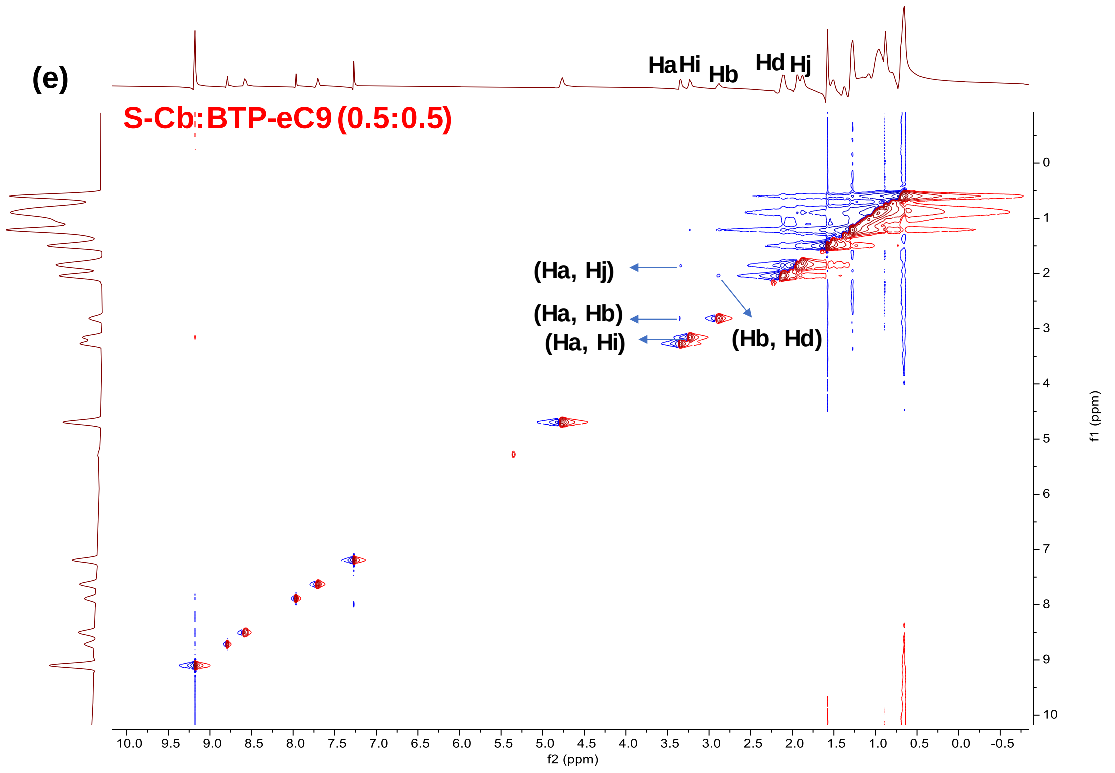

Supplementary Fig. 26 (a) Hydrogen atoms labeling Ha-Hk and their positions on S-Cb, L8-BO, and BTP-eC9. ^{1}H-^{1}H NOSEY NMR spectra of (b) S-Cb; (c) L8-BO, (d) S-Cb:L8-BO (0.5:0.5), and (e) S-Cb:BTP-eC9 (0.5:0.5).

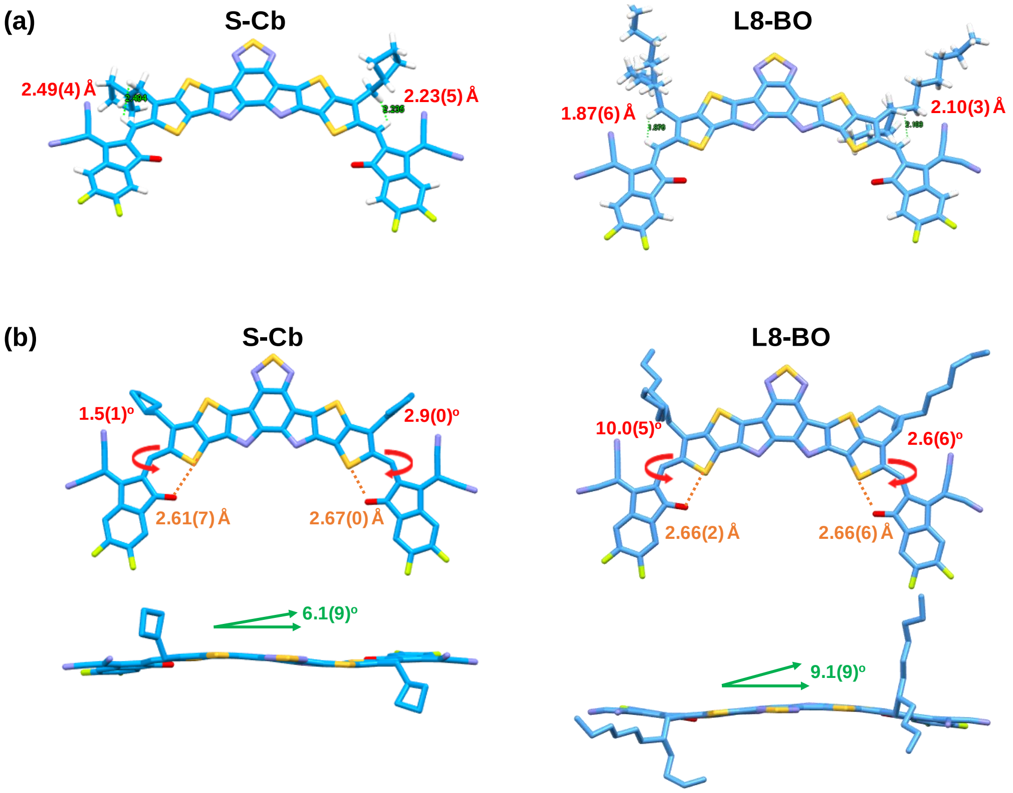

Supplementary Fig. 27 (a) Spatial distance between Hb and He in S-Cb, and Hf and Hh in L8-BO. (b) Molecular planarity of S-Cb and L8-BO.

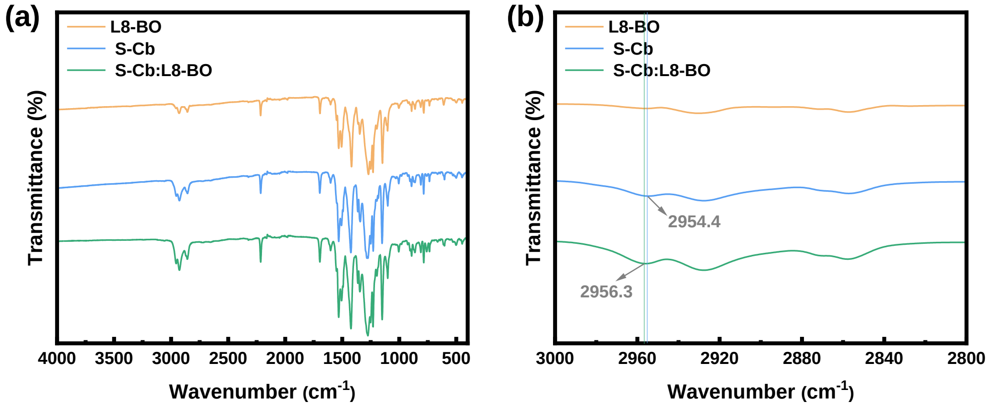

Supplementary Fig. 28 FTIR spectra of L8-BO, S-Cb neat films and S-Cb:L8-BO (1:1) blend film: (a) overall image and (b) local image

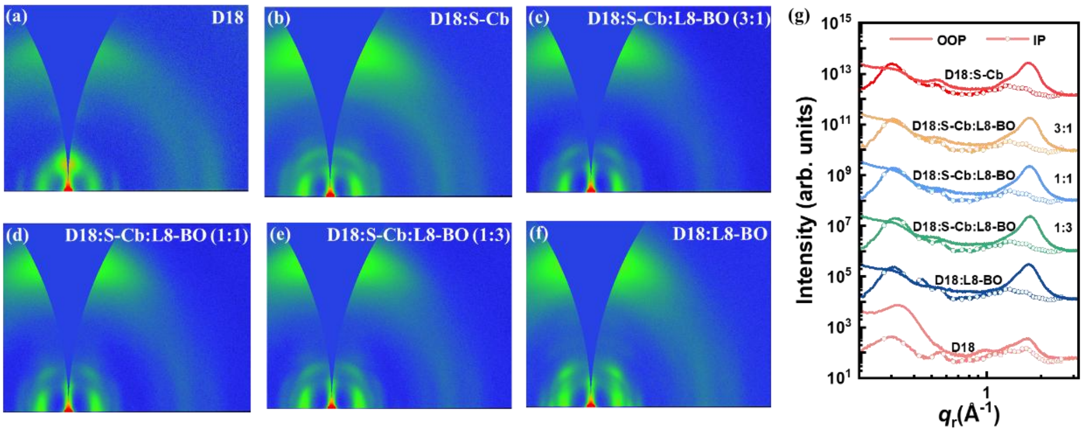

Supplementary Fig. 29 2D GIWAXS patterns: (a) D18; (b) D18:S-Cb; (c) D18:S-Cb:L8-BO (3:1); (d) D18:S-Cb:L8-BO (1:1); (e) D18:S-Cb:L8-BO (1:3); (f) D18:L8-BO. (g) Corresponding 1D-cutting line profiles.

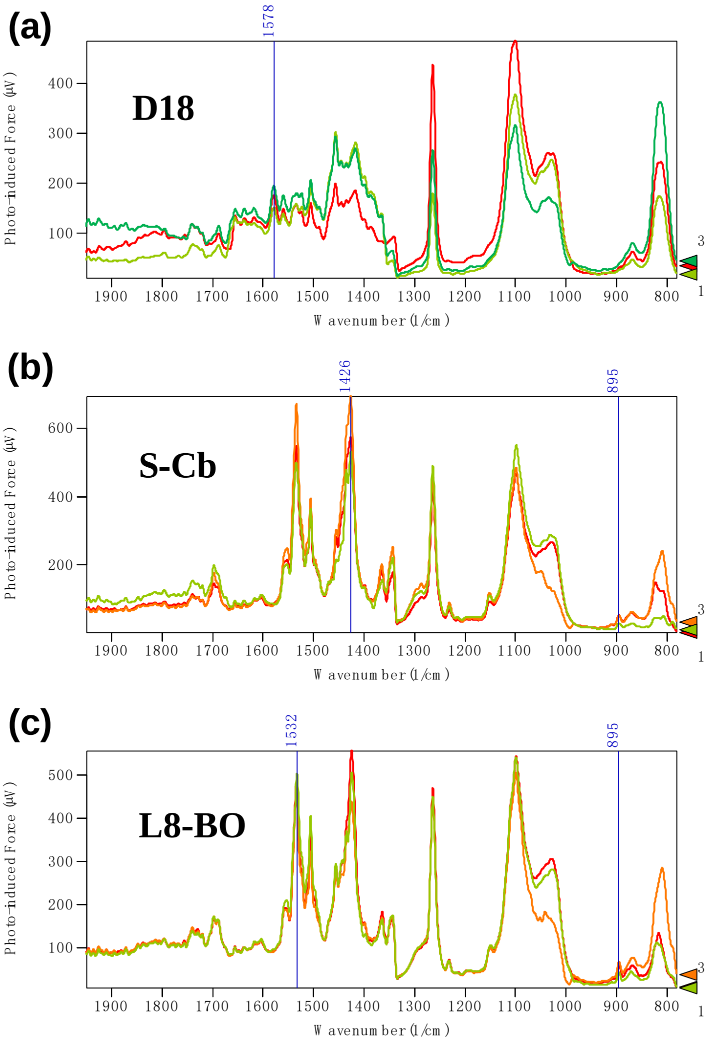

Supplementary Fig. 30 Fourier-transform infrared (FTIR) spectra and characteristic peaks for (a) D18, (b) S-Cb, and (c) L8-BO.

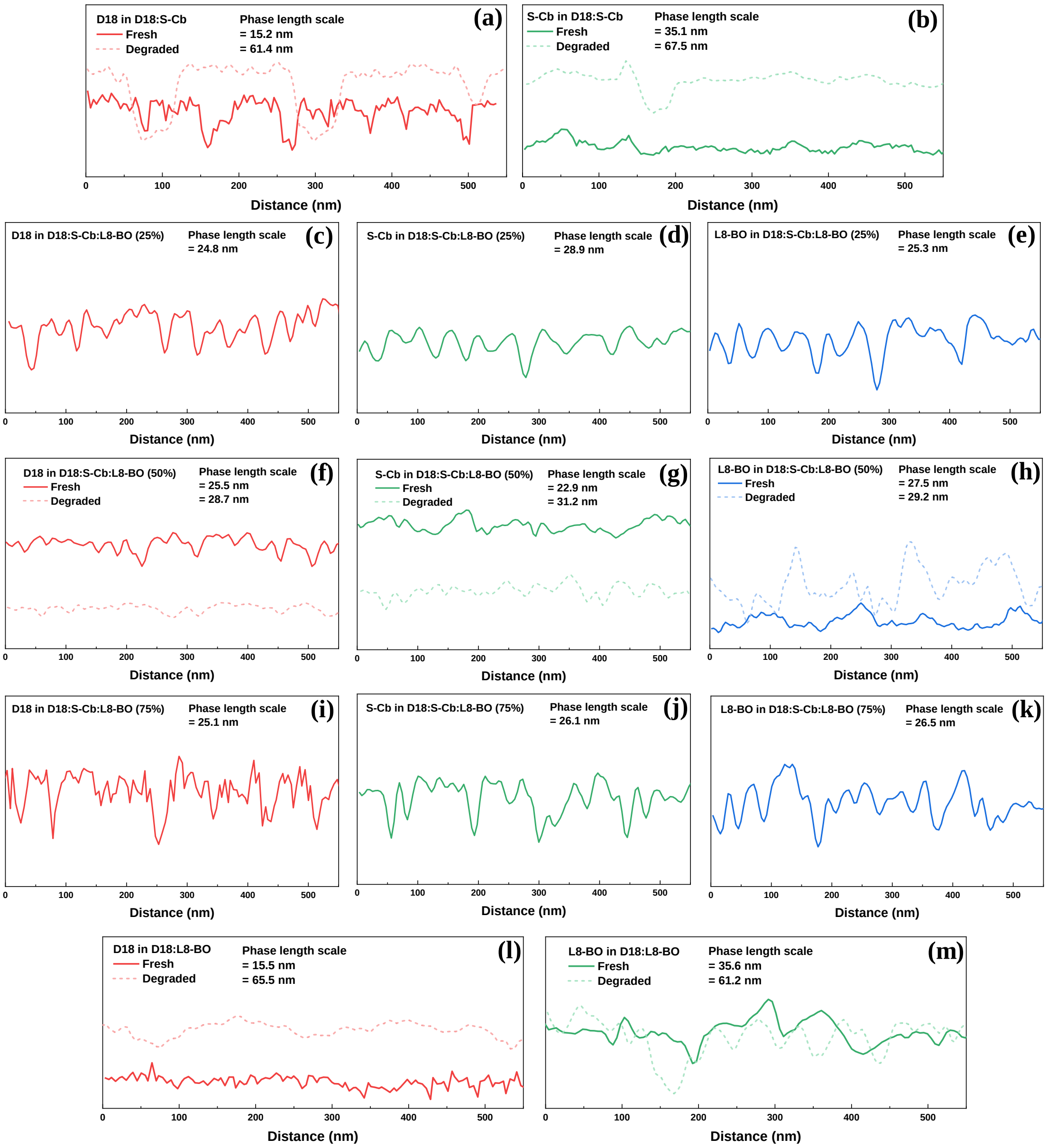

Supplementary Fig. 31 Phase length scales obtained from PiFM measurements: (a-b) D18 and S-Cb components in D18:S-Cb, respectively; (c-e) D18, S-Cb, and L8-BO components in D18:S-Cb:L8-BO (25%), respectively; (f-h) D18, S-Cb, and L8-BO components in D18:S-Cb:L8-BO (50%), respectively; (i-k) D18, S-Cb, and L8-BO components in D18:S-Cb:L8-BO (75%), respectively; (l-m) D18 and L8-BO components in D18:L8-BO, respectively.

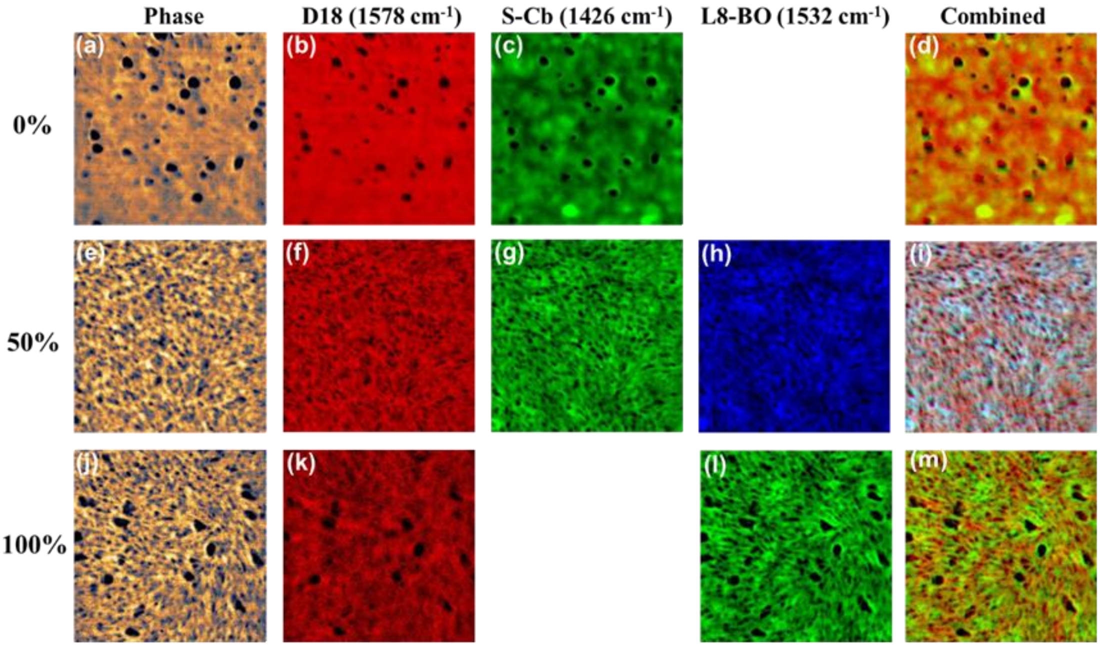

Supplementary Fig. 32 PiFM images of degraded films for (a-d) D18:S-Cb, (e-i) D18:S-Cb:L8-BO (50%), and (j-m) D18:L8-BO.

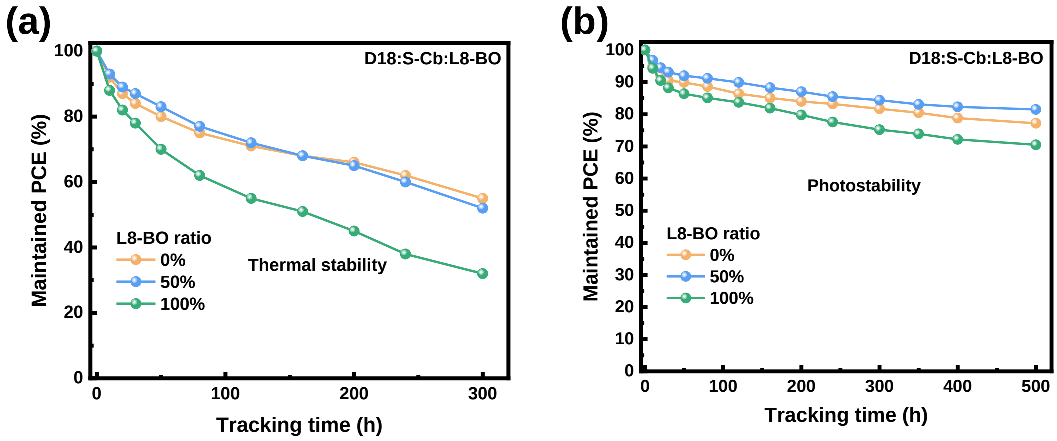

Supplementary Fig. 33 (a) Thermal- and (b) photo-stability test of OSCs based on D18:L8-BO, D18:S-Cb and D18:S-CbLL8-BO (0.5:0.5).

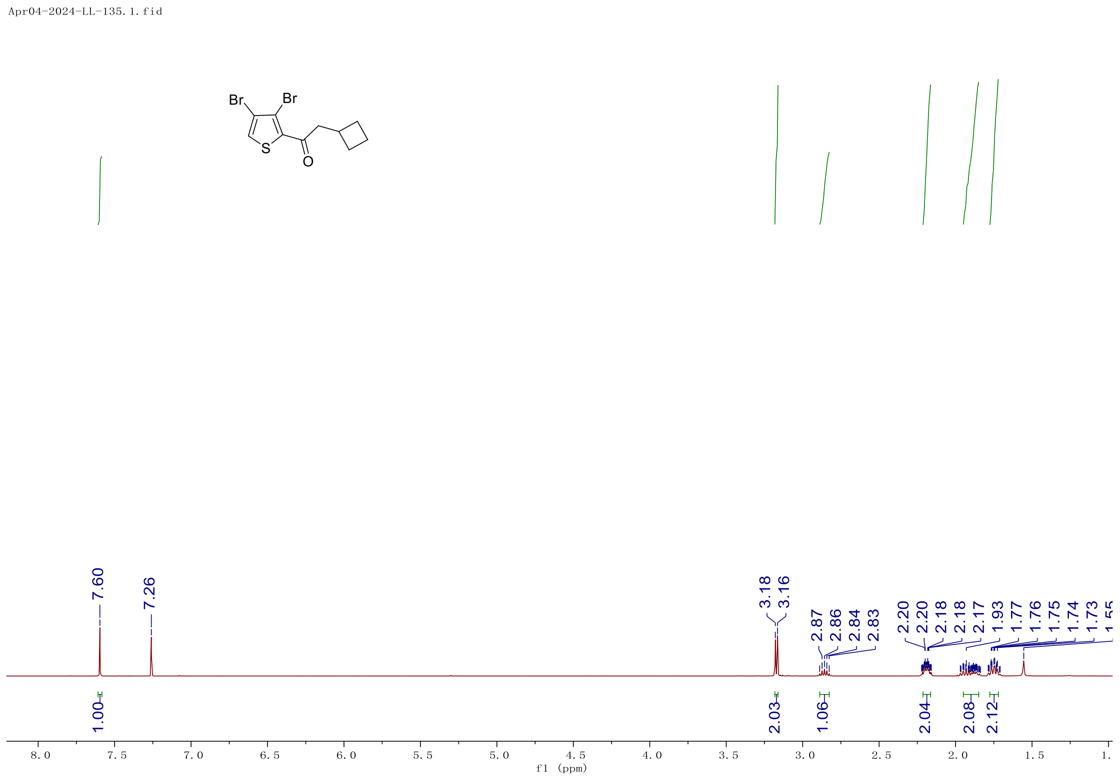

Supplementary Fig. 34 ^{1}H NMR spectrum of **compound 1**.

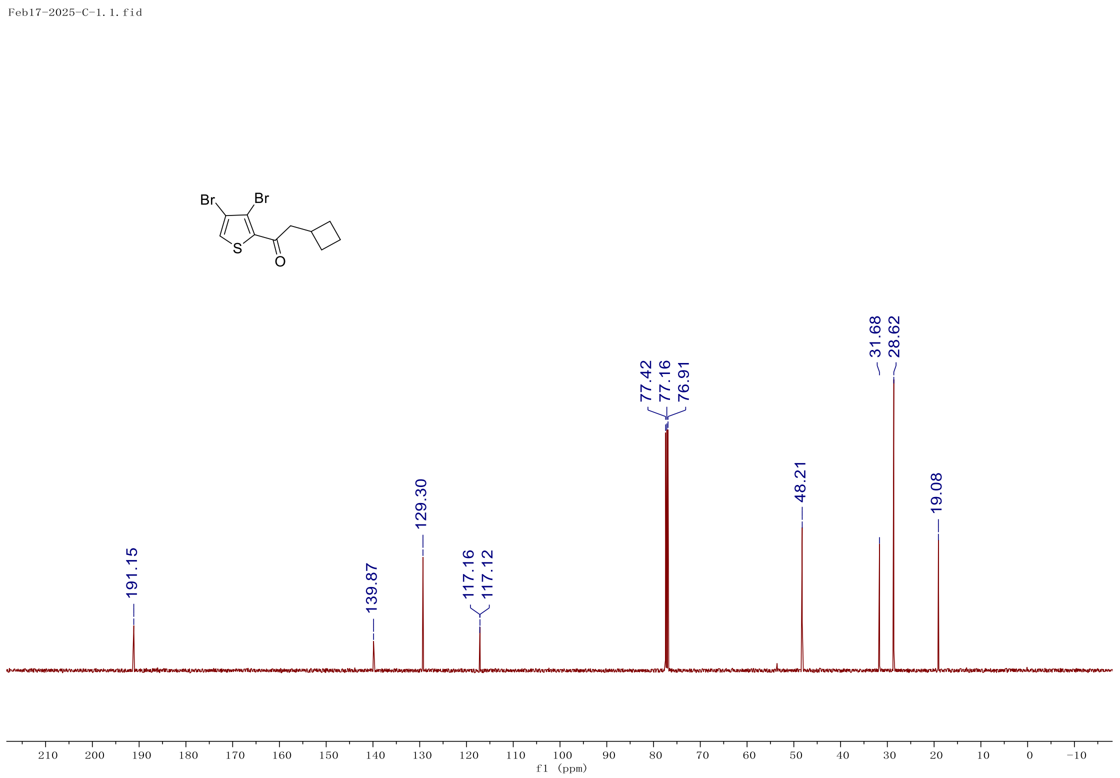

Supplementary Fig. 35 ^{13}C NMR spectrum of **compound 1**.

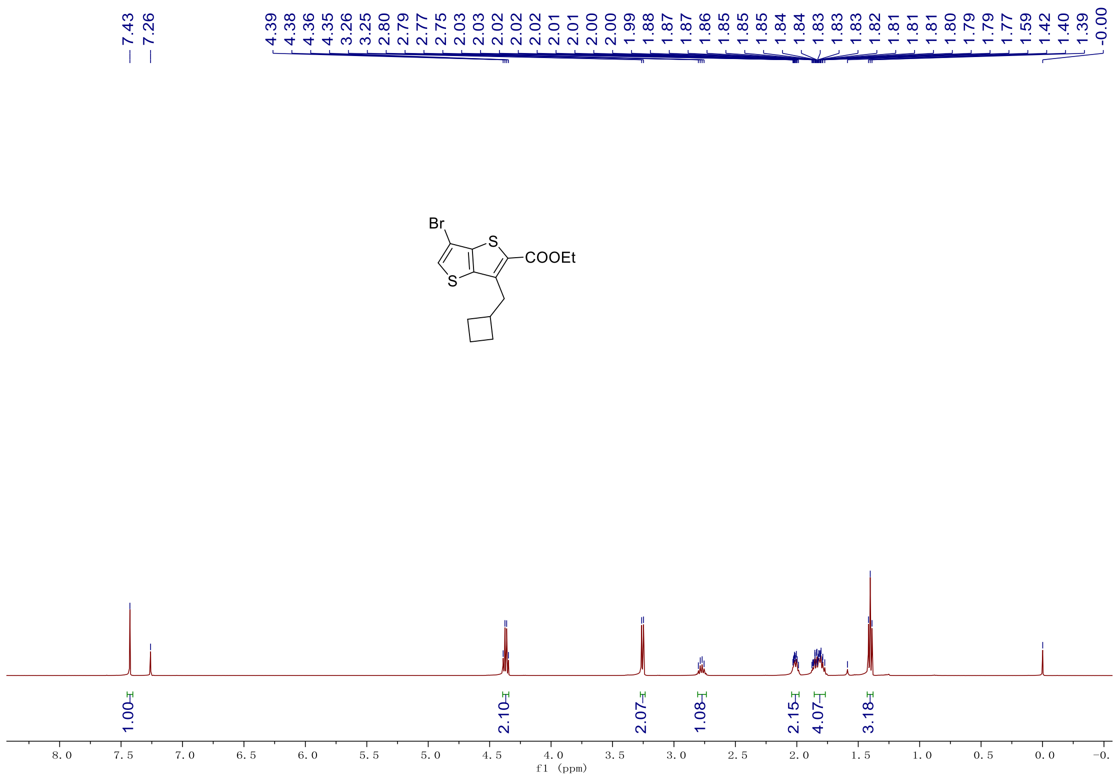

Supplementary Fig. 36 ^{1}H NMR spectrum of **compound 2**.

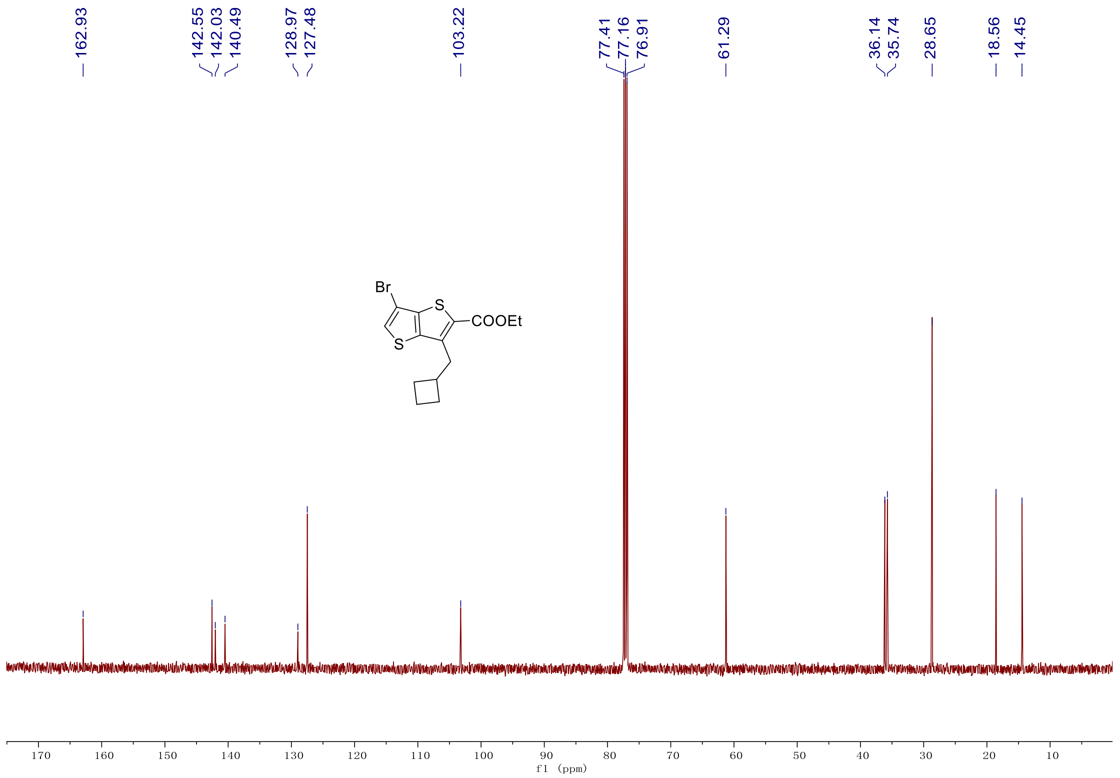

Supplementary Fig. 37 ^{13}C NMR spectrum of **compound 2**.

Apr07-2024-LL-143.1.fid

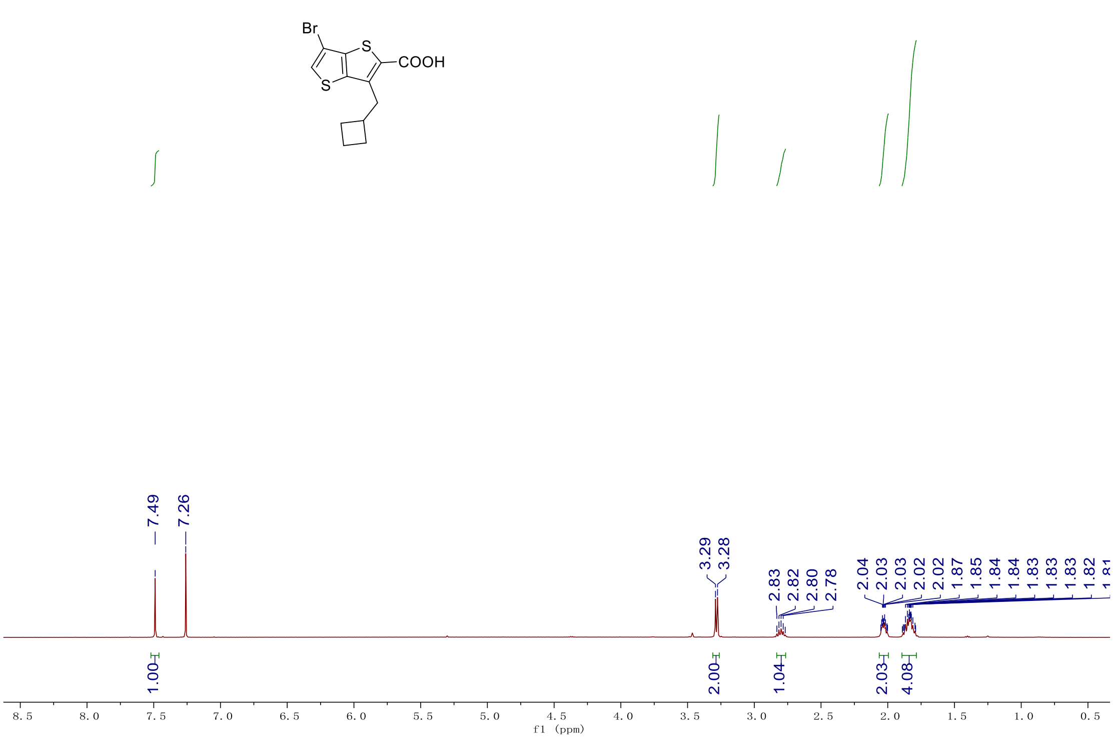

Supplementary Fig. 38 ^{1}H NMR spectrum of **compound 3**.

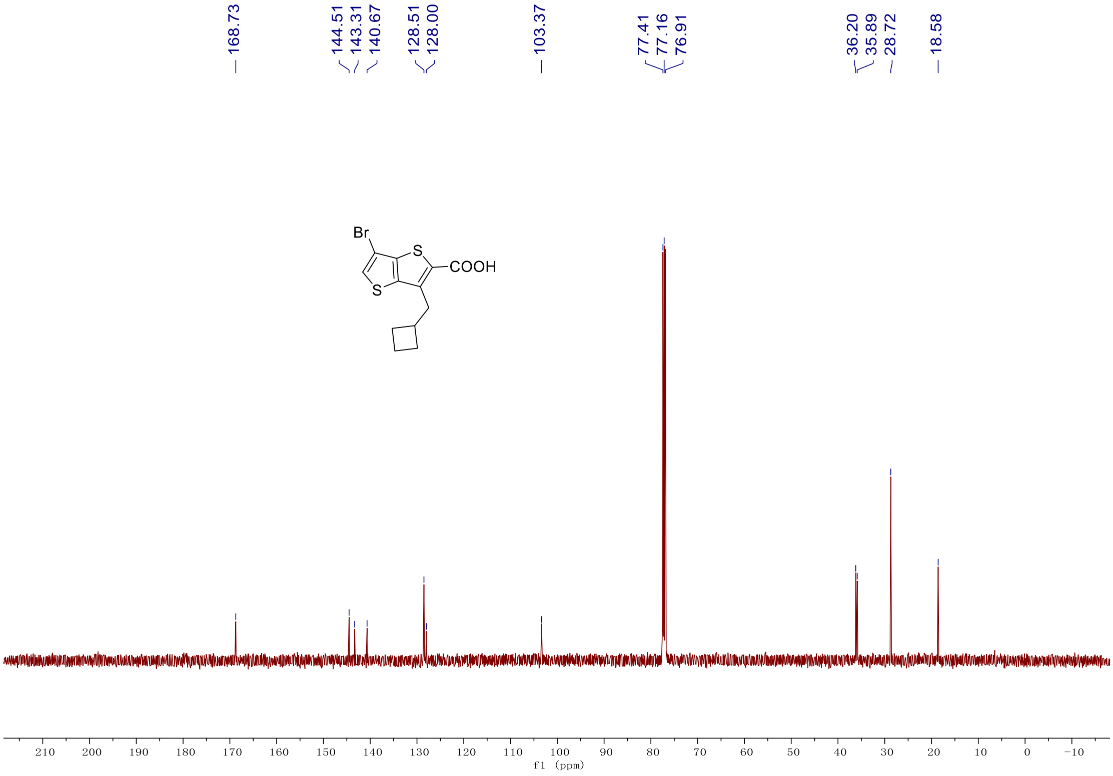

Supplementary Fig. 39 ^{13}C NMR spectrum of **compound 3**.

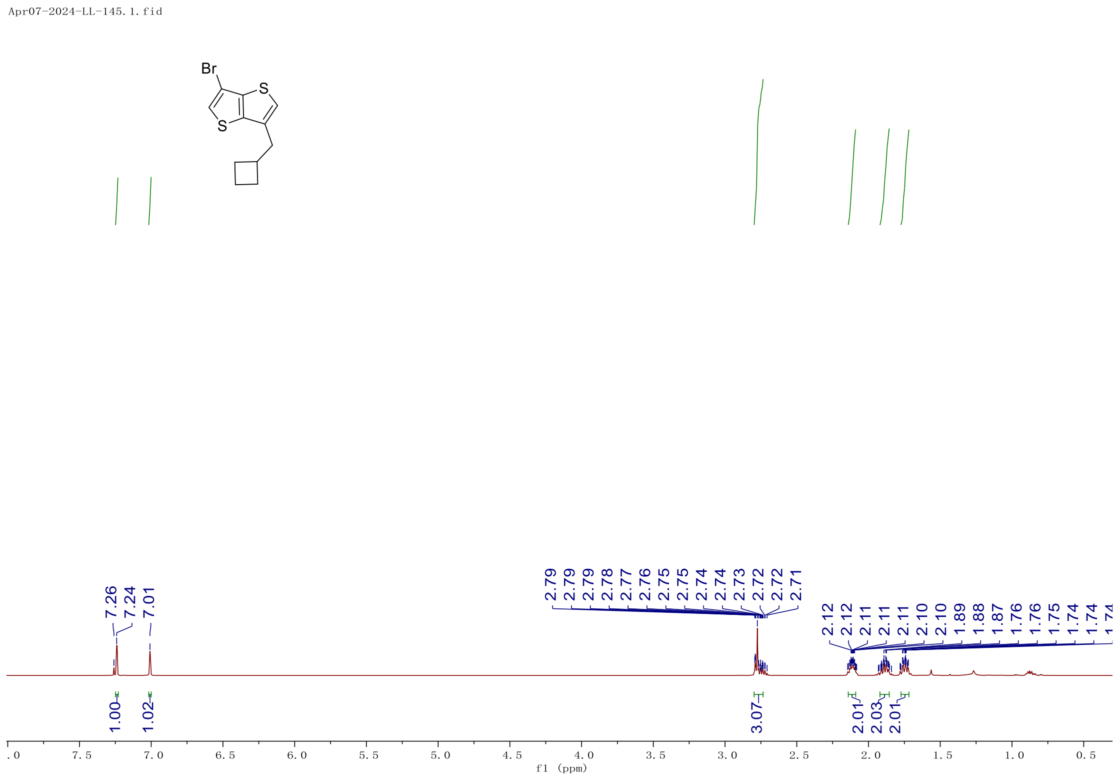

Supplementary Fig. 40 ^{1}H NMR spectrum of **compound 4**.

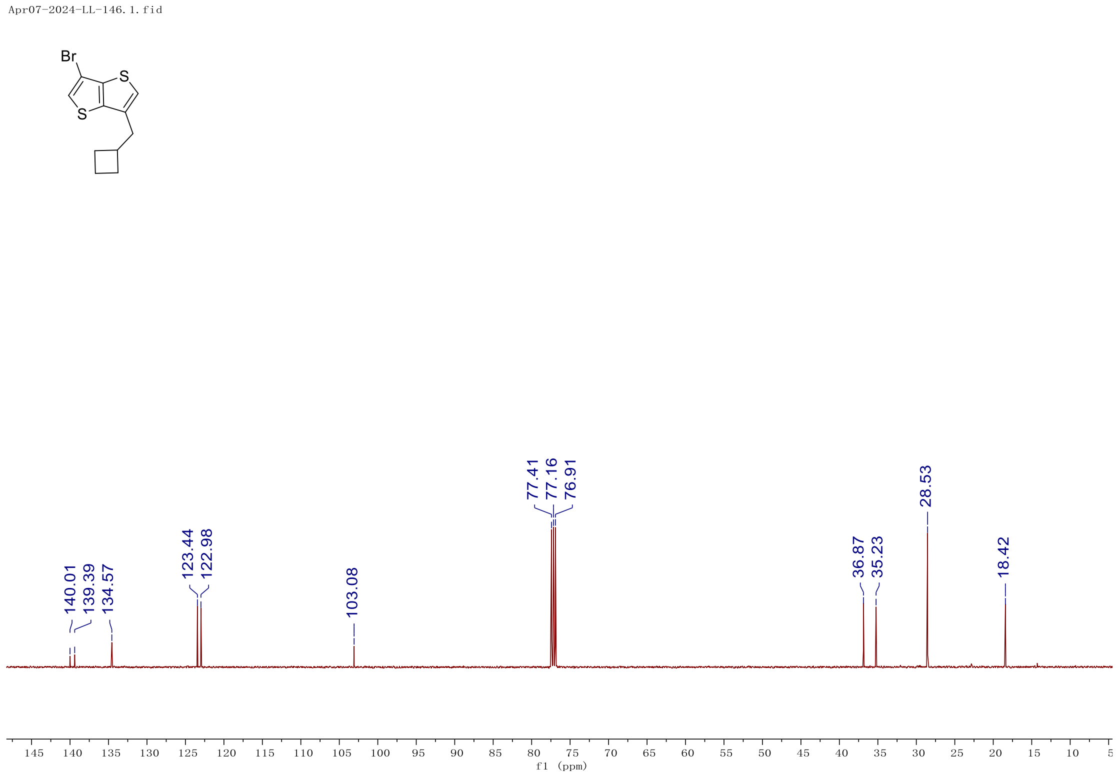

Supplementary Fig. 41 ^{13}C NMR spectrum of **compound 4**.

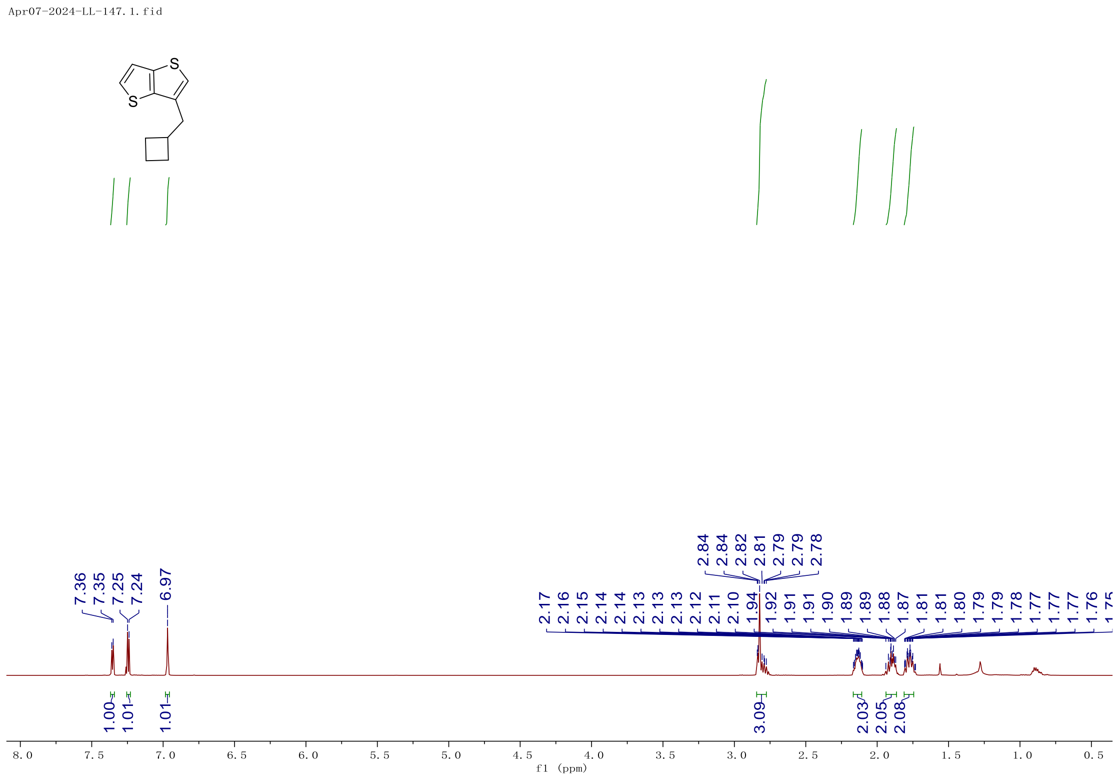

Supplementary Fig. 42 ^{1}H NMR spectrum of **compound 5**.

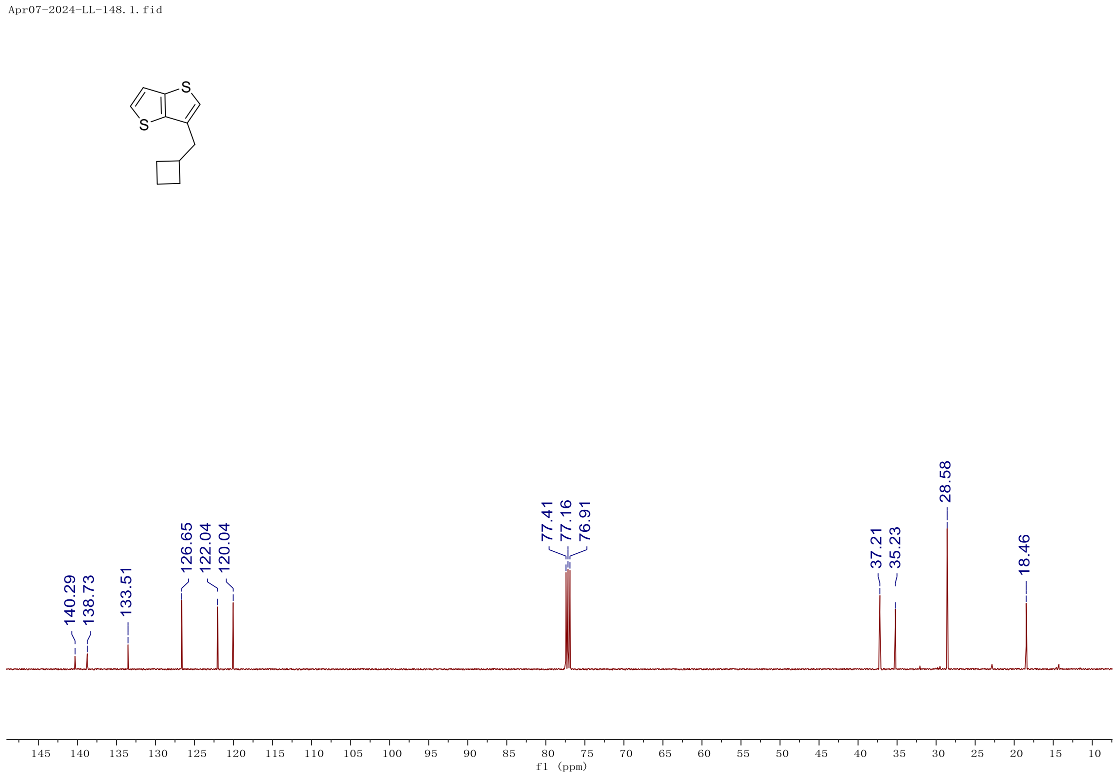

Supplementary Fig. 43 ^{13}C NMR spectrum of **compound 5**.

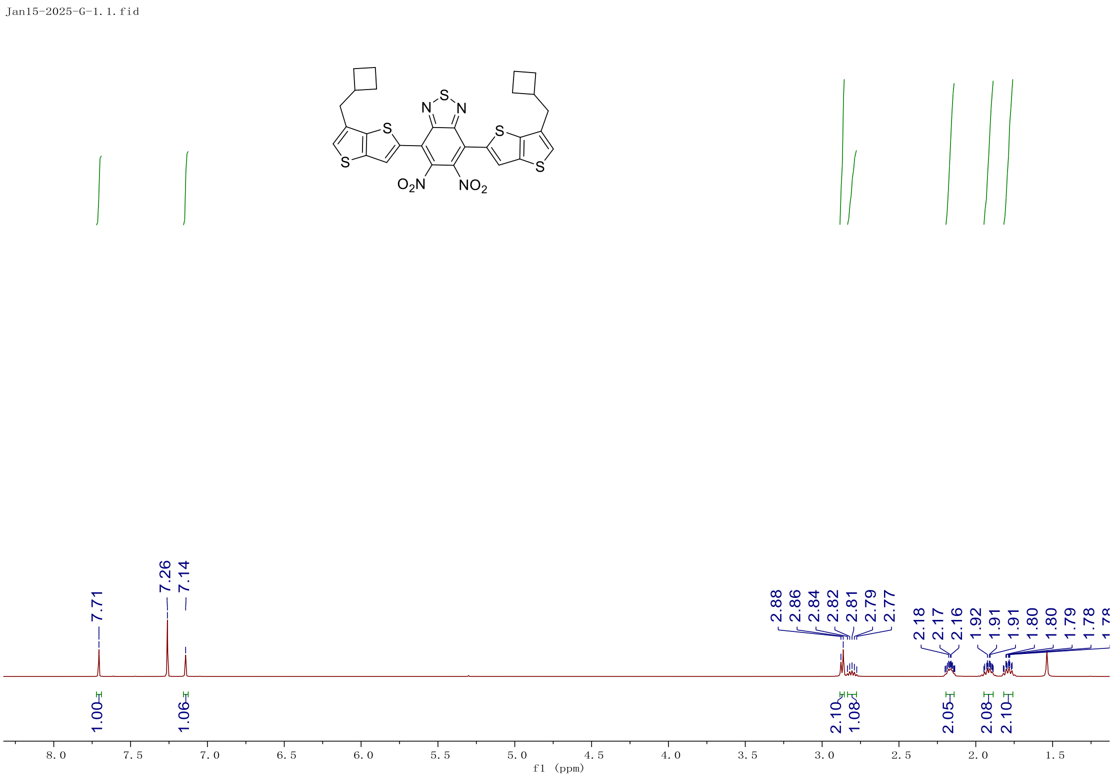

Supplementary Fig. 44 ^{1}H NMR spectrum of **compound 7**.

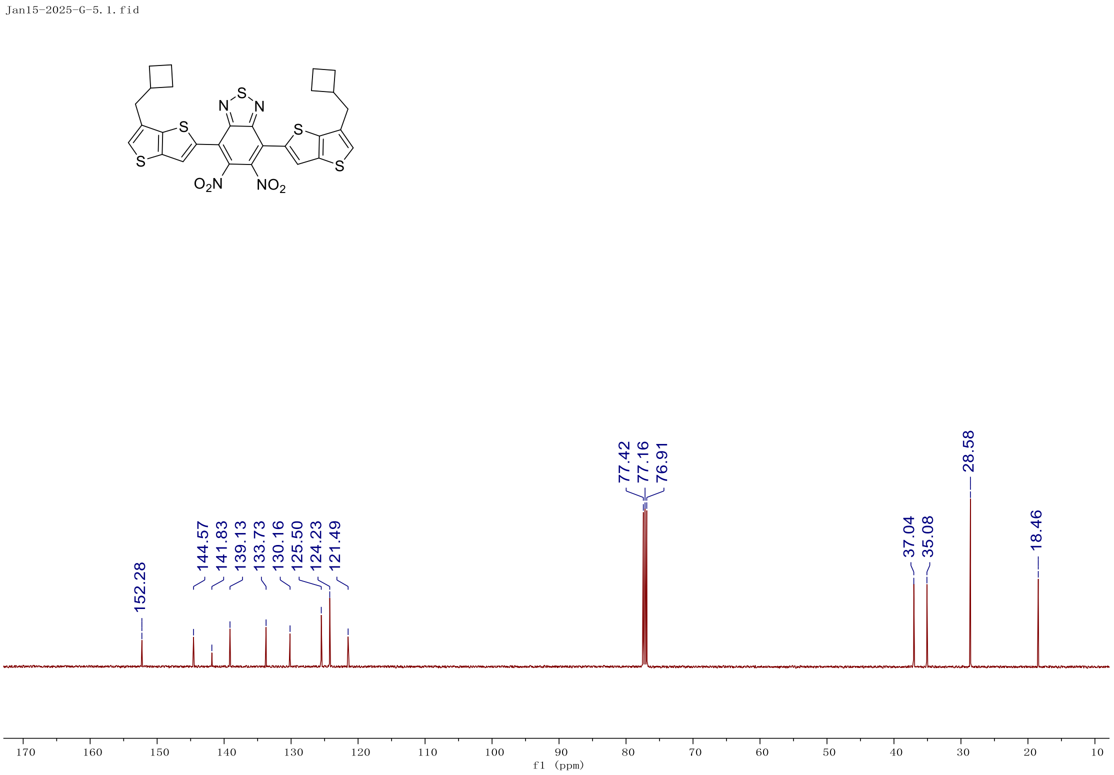

Supplementary Fig. 45 ^{13}C NMR spectrum of **compound 7**.

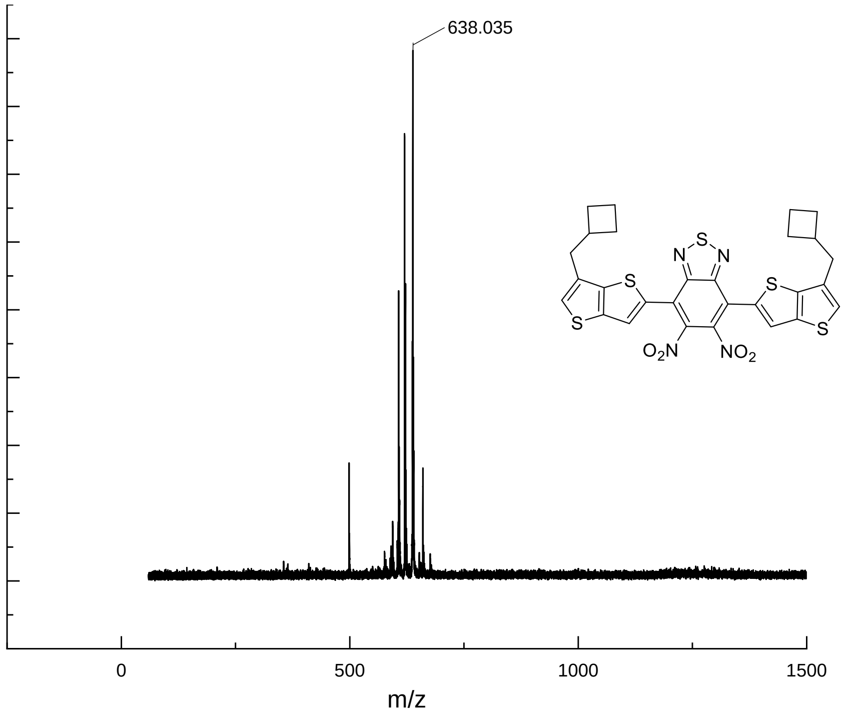

Supplementary Fig. 46 MALDI-TOF-MS spectrum of **compound 7**.

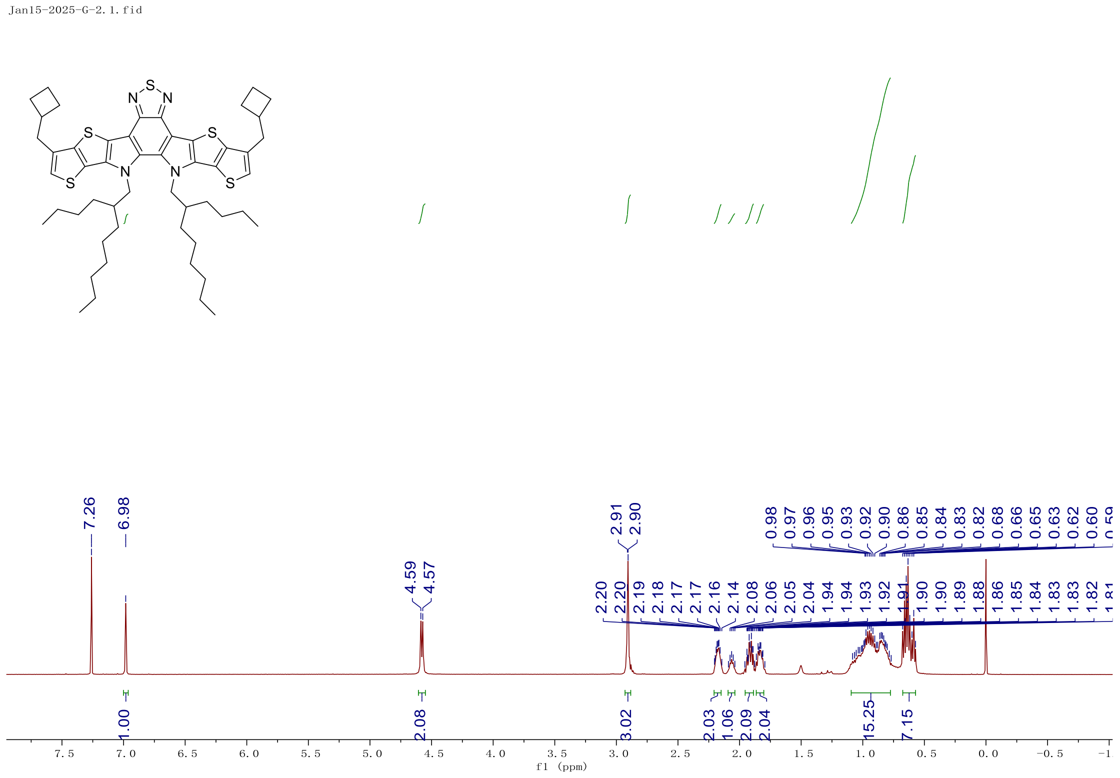

Supplementary Fig. 47 ^{1}H NMR spectrum of **compound 8**.

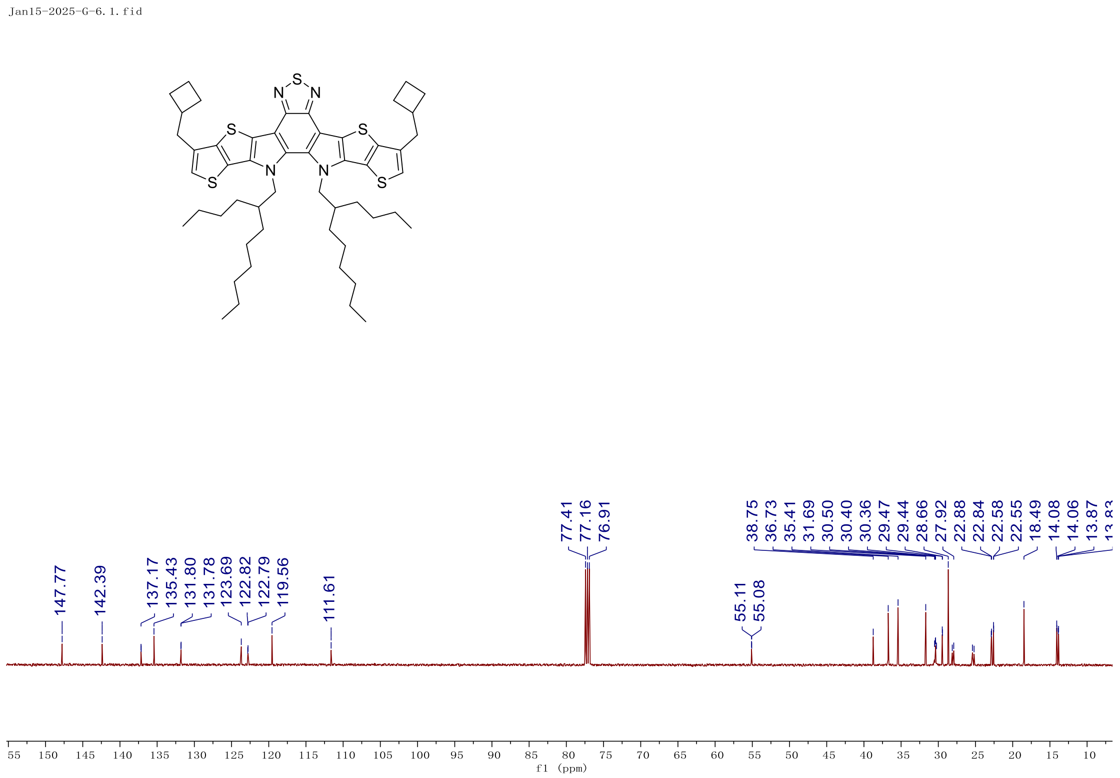

Supplementary Fig. 48 ^{13}C NMR spectrum of **compound 8**.

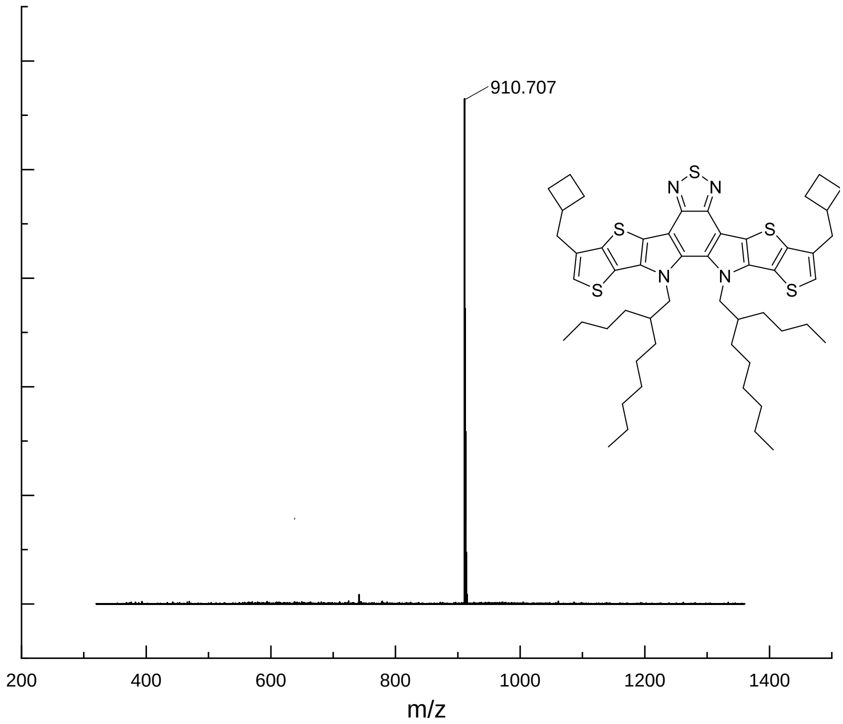

Supplementary Fig. 49 MALDI-TOF-MS spectrum of **compound 8**.

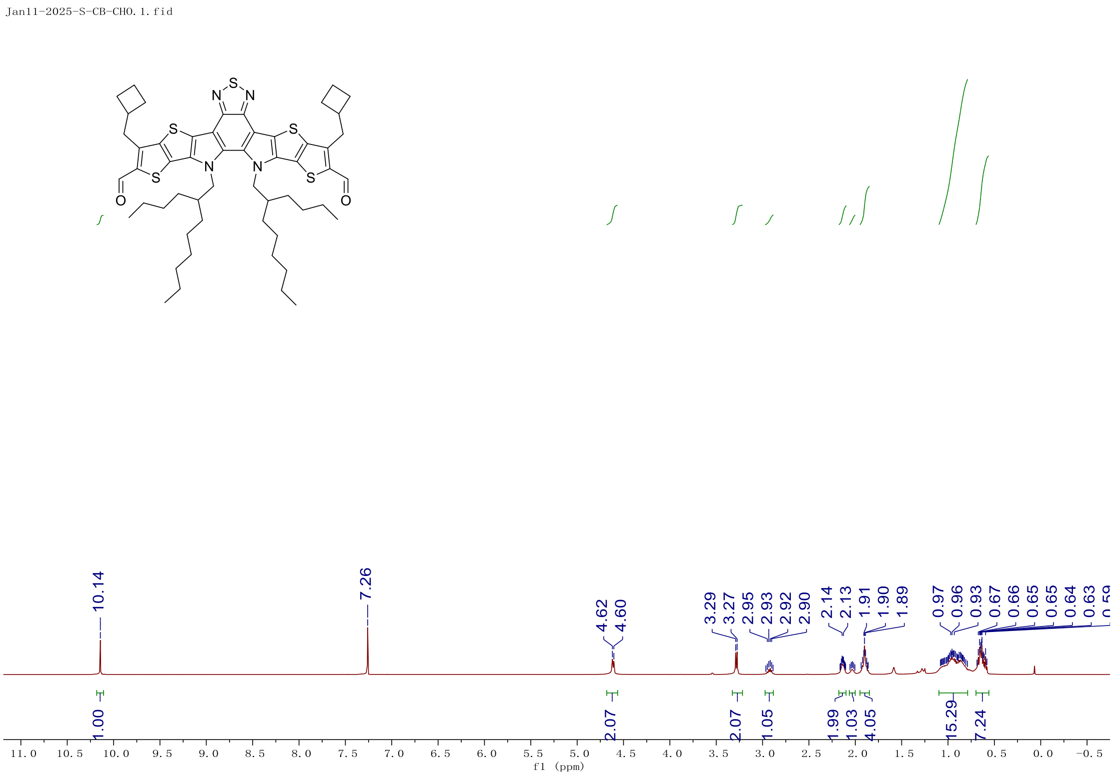

Supplementary Fig. 50 ^{1}H NMR spectrum of **compound 9**.

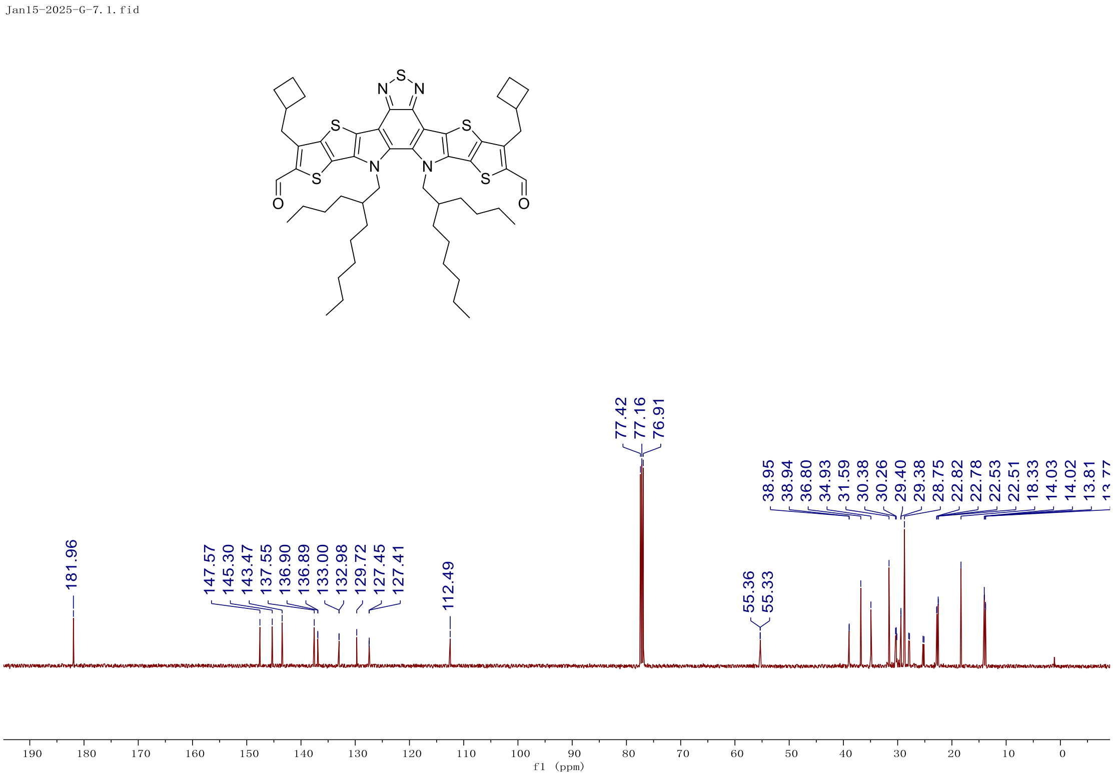

Supplementary Fig. 51 ^{13}C NMR spectrum of **compound 9**.

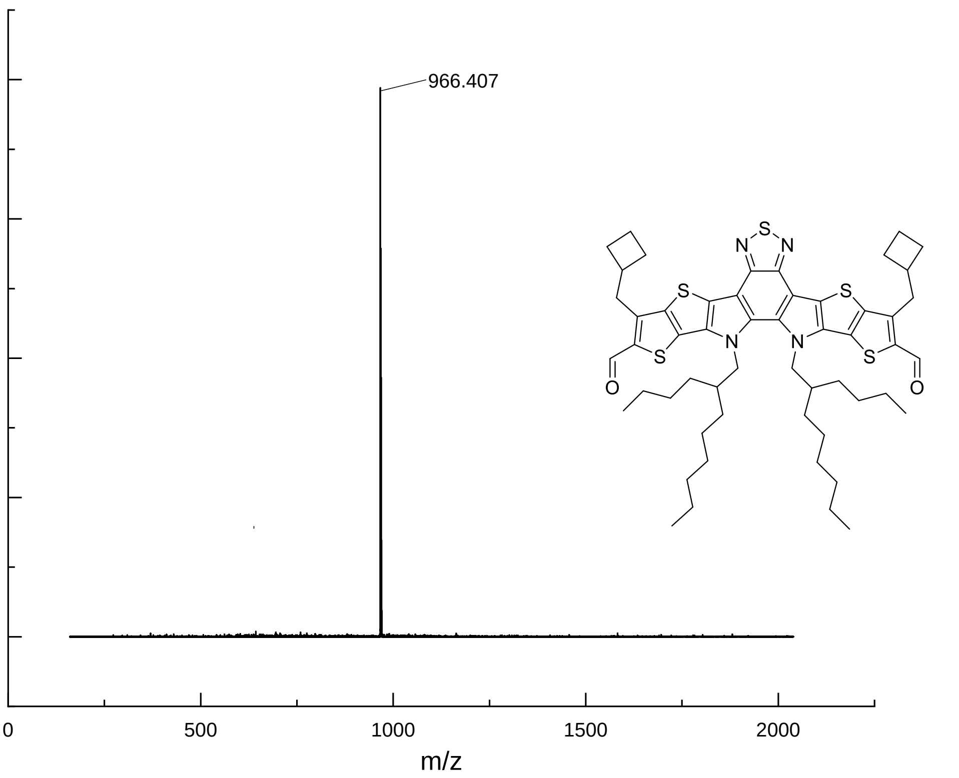

Supplementary Fig. 52 MALDI-TOF-MS spectrum of **compound 9**.

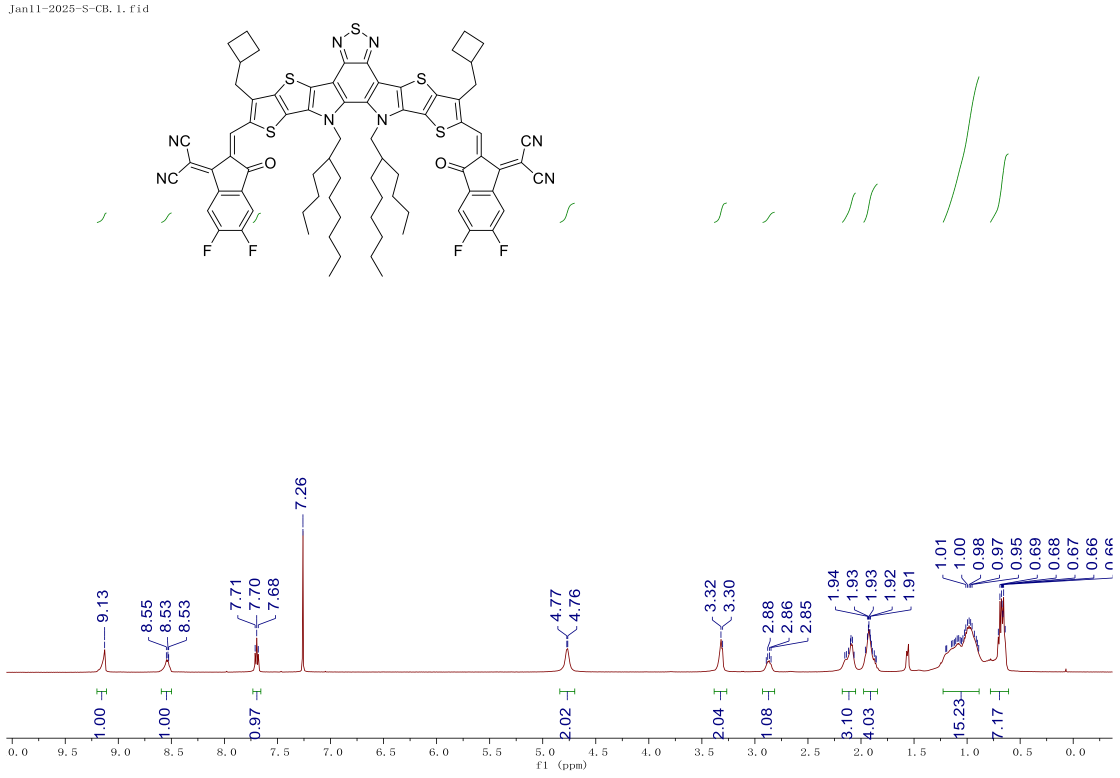

Supplementary Fig. 53 ^{1}H NMR spectrum of **S-Cb**.

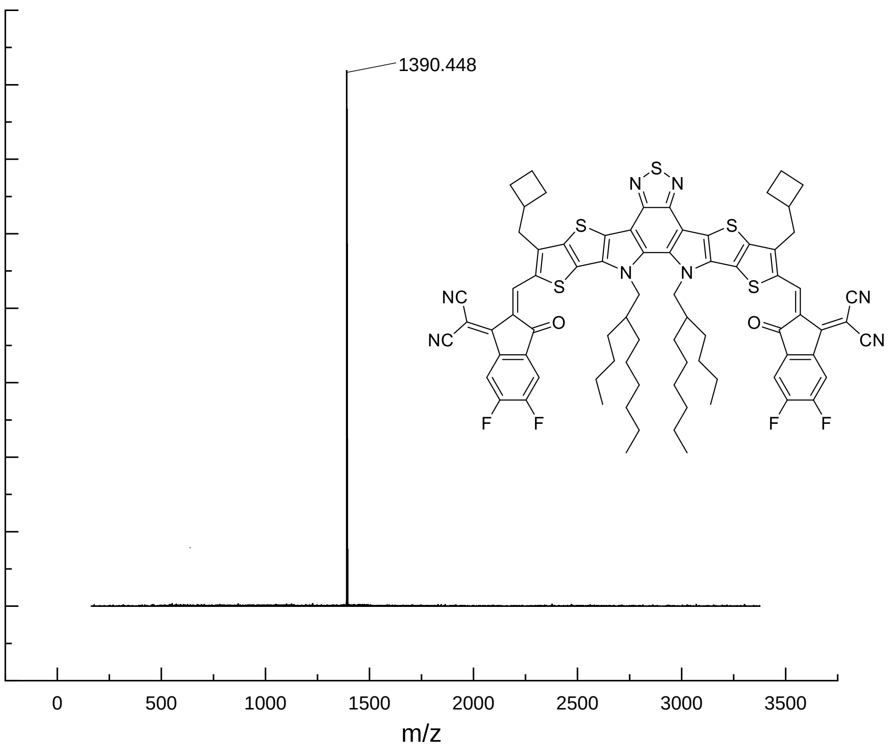

Supplementary Fig. 54 MALDI-TOF-MS spectrum of **S-Cb**.

Supplementary References

1. Lu, T. et al. Independent gradient model based on Hirshfeld partition: A new method for visual study of interactions in chemical systems. *J. Comput. Chem.* **43**, 539-555 (2022).
