# Supplementary material for: Interchain supramolecular interactions drive nearly 21% efficiency organic solar cells
Source: Nat Commun. 2026 Mar 30;17:4590. doi: 10.1038/s41467-026-71199-0 (PMC13195077; doi:10.1038/s41467-026-71199-0)
Supplement: Supplementary file 2 — Reporting Summary [file 41467_2026_71199_MOESM2_ESM.pdf]

nature portfolio

Corresponding author(s): Gang Li

Solar Cells Reporting Summary

Nature Portfolio wishes to improve the reproducibility of the work that we publish. This form is intended for publication with all accepted papers reporting the characterization of photovoltaic devices and provides structure for consistency and transparency in reporting. Some list items might not apply to an individual manuscript, but all fields must be completed for clarity.

For further information on Nature Research policies, including our data availability policy, see Authors & Referees.

Experimental design

Please check the following details are reported in the manuscript, and provide a brief description or explanation where applicable.

1. Dimensions

Area of the tested solar cells

- [x] Yes
- [ ] No

The device contact area was 0.042 cm2, device illuminated area during testing was 0.040 cm2, which was determined by a mask. This information can be found in Devices Fabrications and Characterizations section in Methods.

Explain why this information is not reported/not relevant.

Method used to determine the device area

- [x] Yes
- [ ] No

The area of devices was determined by a mask. This information can be found in Devices Fabrications and Characterizations section in Methods.

Explain why this information is not reported/not relevant.

2. Current-voltage characterization

Current density-voltage (J-V) plots in both forward and backward direction

- [ ] Yes
- [x] No

The hysteresis effect of organic solar cells can be ignored, and the positive and negative scanning results are the same.

Voltage scan conditions

- [x] Yes
- [ ] No

The condition of voltage scanning is forward scanning at a speed of 0.01 V and dwell time of 1 ms.

Explain why this information is not reported/not relevant.

Test environment

- [x] Yes
- [ ] No

The devices were characterized at room temperature in a glove box.

Explain why this information is not reported/not relevant.

Protocol for preconditioning of the device before its characterization

- [ ] Yes
- [x] No

Provide a description of the protocol.

Explain why this information is not reported/not relevant.

Stability of the J-V characteristic

- [ ] Yes
- [x] No

Provide a description of the method used. The stability of the J-V characteristic can be verified with time evolution of the maximum power point or with the photocurrent at maximum power point; see ref. 5 for details.

Explain why this information is not reported/not relevant.

3. Hysteresis or any other unusual behaviour

Description of the unusual behaviour observed during the characterization

- [ ] Yes
- [x] No

Provide a description of hysteresis or any other unusual behaviour observed during the characterization.

Explain why this information is not reported/not relevant.

Related experimental data

- [ ] Yes
- [x] No

Provide a description of the related experimental data.

Explain why this information is not reported/not relevant.

4. Efficiency

External quantum efficiency (EQE) or incident photons to current efficiency (IPCE)

- [x] Yes
- [ ] No

This information can be found in manuscript.

Explain why this information is not reported/not relevant.

A comparison between the integrated response under the standard reference spectrum and the response measure under the simulator

- [x] Yes
- [ ] No

This information can be found in Devices Fabrications and Characterizations section in Methods.

Explain why this information is not reported/not relevant.

For tandem solar cells, the bias illumination and bias voltage used for each subcell

☐ Yes
☒ No

Provide a description of the measurement conditions.

Explain why this information is not reported/not relevant.

5. Calibration

Light source and reference cell or sensor used for the characterization

☒ Yes
☐ No

This information can be found in Devices Fabrications and Characterizations section in Methods.

Explain why this information is not reported/not relevant.

Confirmation that the reference cell was calibrated and certified

☒ Yes
☐ No

This information can be found in Devices Fabrications and Characterizations section in Methods.

Explain why this information is not reported/not relevant.

Calculation of spectral mismatch between the reference cell and the devices under test

☐ Yes
☒ No

Provide a value of the spectral mismatch and/or a description of how it has been taken into account in the measurements.

Explain why this information is not reported/not relevant.

6. Mask/aperture

Size of the mask/aperture used during testing

☒ Yes
☐ No

This information can be found in Devices Fabrications and Characterizations section in Methods.

Explain why this information is not reported/not relevant.

Variation of the measured short-circuit current density with the mask/aperture area

☐ Yes
☒ No

Report the difference in the short-circuit current density values measured with the mask and aperture area.

Explain why this information is not reported/not relevant.

7. Performance certification

Identity of the independent certification laboratory that confirmed the photovoltaic performance

☐ Yes
☒ No

Identify the independent certification laboratory.

Explain why this information is not reported/not relevant.

A copy of any certificate(s)

☐ Yes
☒ No

Certificate copies should be provided in the Supplementary information. Please state the supplementary item number.

Explain why this information is not reported/not relevant.

8. Statistics

Number of solar cells tested

☒ Yes
☐ No

10-15 devices. This information can be found in manuscript.

Explain why this information is not reported/not relevant.

Statistical analysis of the device performance

☒ Yes
☐ No

The average PCE were calculated from 10-15 devices for each OSC. This information can be found in manuscript .

Explain why this information is not reported/not relevant.

9. Long-term stability analysis

Type of analysis, bias conditions and environmental conditions

☒ Yes
☐ No

This information can be found in manuscript.

Explain why this information is not reported/not relevant.
